# Factors Associated with Heritable Pulmonary Arterial Hypertension Exert Convergent Actions on the miR-130/301-Vascular Matrix Feedback Loop

**DOI:** 10.3390/ijms19082289

**Published:** 2018-08-04

**Authors:** Thomas Bertero, Adam L. Handen, Stephen Y. Chan

**Affiliations:** 1 Université Côte d’Azur, CNRS UMR7284, INSERM U1081, IRCAN, Nice 06100, France; 2 Center for Pulmonary Vascular Biology and Medicine, Pittsburgh Heart, Lung, Blood, and Vascular Medicine Institute, Division of Cardiology, Department of Medicine, University of Pittsburgh School of Medicine and University of Pittsburgh Medical Center, Pittsburgh, PA 15213, USA; handena@pitt.edu

**Keywords:** heritable pulmonary hypertension, pulmonary arterial hypertension, genetic predisposition, microRNA, extracellular matrix, fibrosis

## Abstract

Pulmonary arterial hypertension (PAH) is characterized by occlusion of lung arterioles, leading to marked increases in pulmonary vascular resistance. Although heritable forms of PAH are known to be driven by genetic mutations that share some commonality of function, the extent to which these effectors converge to regulate shared processes in this disease is unknown. We have causally connected extracellular matrix (ECM) remodeling and mechanotransduction to the miR-130/301 family in a feedback loop that drives vascular activation and downstream PAH. However, the molecular interconnections between factors genetically associated with PAH and this mechano-driven feedback loop remain undefined. We performed systematic manipulation of matrix stiffness, the miR-130/301 family, and factors genetically associated with PAH in primary human pulmonary arterial cells and assessed downstream and reciprocal consequences on their expression. We found that a network of factors linked to heritable PAH converges upon the matrix stiffening-miR-130/301-PPARγ-LRP8 axis in order to remodel the ECM. Furthermore, we leveraged a computational network biology approach to predict a number of additional molecular circuits functionally linking this axis to the ECM. These results demonstrate that multiple genes associated with heritable PAH converge to control the miR-130/301 circuit, triggering a self-amplifying feedback process central to pulmonary vascular stiffening and disease.

## 1. Introduction

Pulmonary hypertension (PH) and a particularly severe subtype, pulmonary arterial hypertension (PAH), are characterized by a complex panvasculopathy involving excessive proliferation and dysregulation of multiple cell types, as well as inflammation and fibrosis throughout the vasculature, leading to increased pulmonary arterial pressure and adverse remodeling of the pulmonary arterioles and often the right ventricle [1]. Exogenous injury (i.e., hypoxia, inflammation, infection) and various other illnesses are linked to PH development, and a growing list of mutations in specific pathogenic genes predispose to hereditary forms of PH [2]. Etiologies of PH are numerous, and five types of PH have been defined in an official World Health Organization (WHO) classification system [3], most recently revised in 2018. WHO Group 1 consists of individuals suffering from PAH, stemming either from idiopathic and hereditary forms or secondarily from co-morbidities such as congenital heart disease, autoimmune disease, drug and toxin exposure, and infections. WHO Groups 2–5 can be triggered by a wide range of other conditions, such as left heart disease, chronic hypoxia, and thromboembolic disease, among others. PH and particularly PAH are highly morbid conditions.

Haploinsufficient loss-of-function mutations in the bone morphogenetic protein type 2 receptor (*BMPR2*) gene, a member of the transforming growth factor-beta (TGF-β) superfamily, account for over 80% of hereditary PAH cases and approximately 20% of idiopathic PAH cases [4]. Several mutations in other genes have been reported, accounting for at least a portion of the remainder of PAH heritability. Pathogenic mutations have been identified in genes encoding other components of the TGF-β/bone morphogenetic protein (BMP) signaling pathways, including *ACVRL1*, *ENG*, *GDF2* [5], *SMAD9*, and *CAV1*, providing compelling evidence for a central role of dysregulated BMP signaling in PAH pathogenesis [2]. More recently, mutations in genes not directly linked to TGF-β/BMP signaling, including *KCNK3* [6], *TBX4* [7], as well as *AQP1* [5], *ATP13A3* [5], *GCN2* [8], and *SOX17* [5] have been linked to PAH; many of these mutations were identified by whole genome sequencing. Moreover, a locus in the *CBLN2* gene was linked to PAH using a genome-wide association study [9]. Yet, beyond their immediate relation to TGF-β/BMP signaling for some, contribution of these genetic factors to PAH progression, particularly in relation to one another, remains incompletely described [2].

Recently, we [10] and others [11] observed that vascular stiffness and extracellular matrix (ECM) remodeling promotes pulmonary vascular dysfunction early in disease pathogenesis. In general, ECM remodeling is a complex process, governed through a balance between ECM protein deposition and degradation, as well as ECM assembly via collagen crosslinking enzymes such as lysyl oxidase (LOX). Decreased vessel wall compliance (i.e., stiffness) occurs in various forms of PH and is an important contributor and index of disease progression [12,13,14,15,16]. Vascular stiffness is determined by vascular tone and the quantity and composition of the ECM. While regulators of vascular tone have been very well studied, less is known about effectors and mechanisms that might regulate vascular stiffness by modulating ECM production/composition. Although anatomic location may dictate distinct effects on disease progression, stiffness in both the proximal and distal pulmonary arterial tree is important for overall pathogenesis [11] but the molecular mechanisms controlling these processes are only beginning to be appreciated.

The structural alterations in the pulmonary vasculature are controlled by activated vascular cells, which in PAH exhibit metabolic reprogramming and consequent hyperproliferative, migratory, and invasive capabilities. Given their inherent pleiotropic actions to repress multiple gene targets simultaneously, microRNAs (miRNAs) are essential mediators of multiple cellular processes and may be ideal candidates to provide comprehensive and integrated control of PAH. Previously, we utilized a network-based approach to investigate the role of miRNAs in the integrated control of PH pathogenesis and described the proliferative and vasoconstrictive actions of miR-130/301 family in PH [17,18]. Beyond these functions, we also reported a prominent component of their related gene targets associated with ECM biology. We established vascular matrix stiffness as an early, pervasive driver of many types of PH [10], controlled by mechanoactivation of two related transcriptional co-activators, YAP (Yes Associated Protein 1) and TAZ (Transcriptional Coactivator with PDZ-Binding Motif), factors that are known to regulate proliferation, survival, organ size, and the ECM. We found that downstream induction of the microRNA-130/301 family further engages a network of related gene targets including the PPARγ-APOE-LRP8 axis that regulates LOX activity to coordinate ECM remodeling and sustain matrix stiffening in PH. Yet, whether the actions of these seemingly disparate factors that are genetically associated with PAH converge to activate this YAP/TAZ-miR-130/301 feedback loop remains undefined. Consequently, in this study we investigated whether factors that are genetically associated with PAH can influence the miR-130/301-ECM remodeling feedback loop, as well as whether the miR-130/301-ECM remodeling feedback loop controls expression of those same factors.

## 2. Results

### 2.1. Matrix Stiffening Modulates the Expression of Several Factors Genetically Associated with PAH

**Figure 1:**
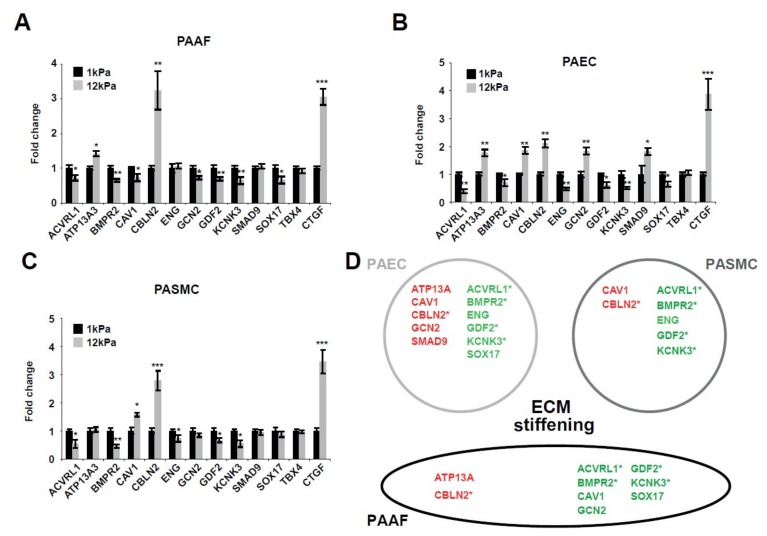
Factors genetically associated with PAH are responsive to matrix stiffening. (**A**–**C**) Pulmonary arterial cells were plated on soft (1 kPa) or stiff (12 kPa) hydrogel for 48 h. Expression level of factors genetically associated with PAH in PAAFs (**A**), PAECs (**B**), and PASMCs (**C**) were analyzed by RT-qPCR. Mean expression in control groups (1 kPa) was assigned a fold change of 1, to which relevant samples were compared. Data are expressed as mean ± SD (* *p* < 0.05, ** *p* < 0.01, *** *p* < 0.001) of at least three independent experiments. Paired samples were compared using a 2-tailed Student’s *t*-test. (**D**) Schematic of the main results. Red: upregulated genes; Green: downregulated genes; * denote consistent and significant modulation in the three vascular cell types.

Since factors with specific genetic mutations drive hereditary PAH, and since matrix stiffening is an early driver of this disease, we hypothesized that ECM stiffening also controls the expression of these factors. To determine whether mechanical or physical cues conveyed by ECM stiffness modulate the expression of these factors, we cultivated pulmonary arterial adventitial fibroblasts (PAAFs), endothelial cells (PAECs), and smooth muscle cells (PASMCs) on ECM representing physiologic (soft; 1 kPa) or pathophysiologic (stiff; 12 kPa) levels of vascular stiffness [19]. By RT-qPCR, transcript expression levels of genes associated with PAH were quantified (Figure 1). Matrix stiffening significantly increased CBLN2 and decreased *ACVRL1*, *BMPR2*, *GDF2*, and *KCNK3* across all vascular cell types tested: PAAFs (Figure 1A), PAECs (Figure 1B), and PASMCs (Figure 1C). Conversely, cell type context-specificity was noted in the alterations of other genes. This was the case for *GCN2*, a gene related to the amino acid stress response pathway, which was downregulated in PAAFs, but upregulated in PAECs, by stiff matrix. Alternatively, in PAECs and PASMCs, stiff matrix upregulated *CAV1* but downregulated this gene in PAAFs (Figure 1D). 

In sum, the majority (10 out of 12) of tested factors were found to be sensitive to mechanical cues conveyed by the ECM, suggesting that, in addition to genetic mutation, the levels of these PAH drivers are dynamically regulated by biophysical triggers. 

### 2.2. The miR-130/301 Family is Modulated by Several Factors Genetically Associated with PAH

**Figure 2:**
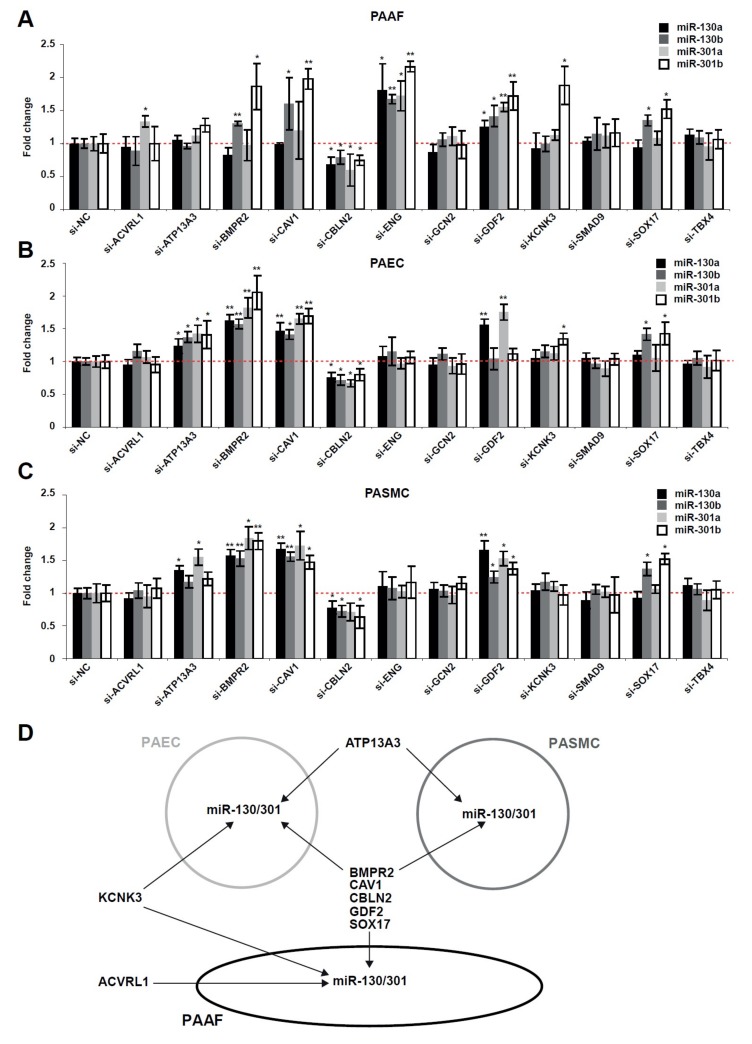
Factors genetically associated with PAH modulate the expression of miR-130/301 family members. (**A**–**C**) Following transfection with the indicated siRNA, transcript levels of miR-130/301 members were analyzed by RT-qPCR in PAAFs (**A**), PAECs (**B**), and PASMCs (**C**). Mean expression of a given miRNA in the control group (si-NC) was assigned a fold change of 1, to which corresponding miRNA levels in experimental groups were compared. Data are expressed as mean ± SD (* *p* < 0.05, ** *p* < 0.01) of three independent experiments. One-way ANOVA and post-hoc Tukey tests were used for group comparisons. (**D**) Schematic of the main results.

Because matrix stiffness modulates genes linked to hereditary PAH (Figure 1) as well as miR-130/301 family members [10], we wanted to determine whether these factors genetically associated with PAH control expression of the miR-130/301 family (Figure 2). First, by RT-qPCR, we confirmed the robust knockdown of these factors after specific siRNA sequence transfection in human PAAFs, PAECs, and PASMCs (Figure S1). Adding to findings from our original published report [18], such knockdown of *BMPR2*, *CAV1*, *GDF2*, and *SOX17* in turn increased miR-130/301 family member expression, while inhibition of CBLN2 decreased miR-130/301 family members in human PAAFs (Figure 2A), PAECs (Figure 2B), and PASMCs (Figure 2C). In addition, we found that *KCNK3* knockdown increased miR-130/301 family expression in both PAAFs and PAECs, while knockdown of *ENG* and *ACVRL1* increased miR-130/301 members only in PAAFs. Moreover, ATP13A3 knockdown increased miR-130/301 members in both PAECs and PASMCs.

Together, these results indicate the broad convergence of genetically associated PAH factors on coordinated regulation of this key microRNA family (Figure 2D).

### 2.3. The miR-130/301 Family Controls the Vascular Expression of Factors Associated with Hereditary PAH

**Figure 3:**
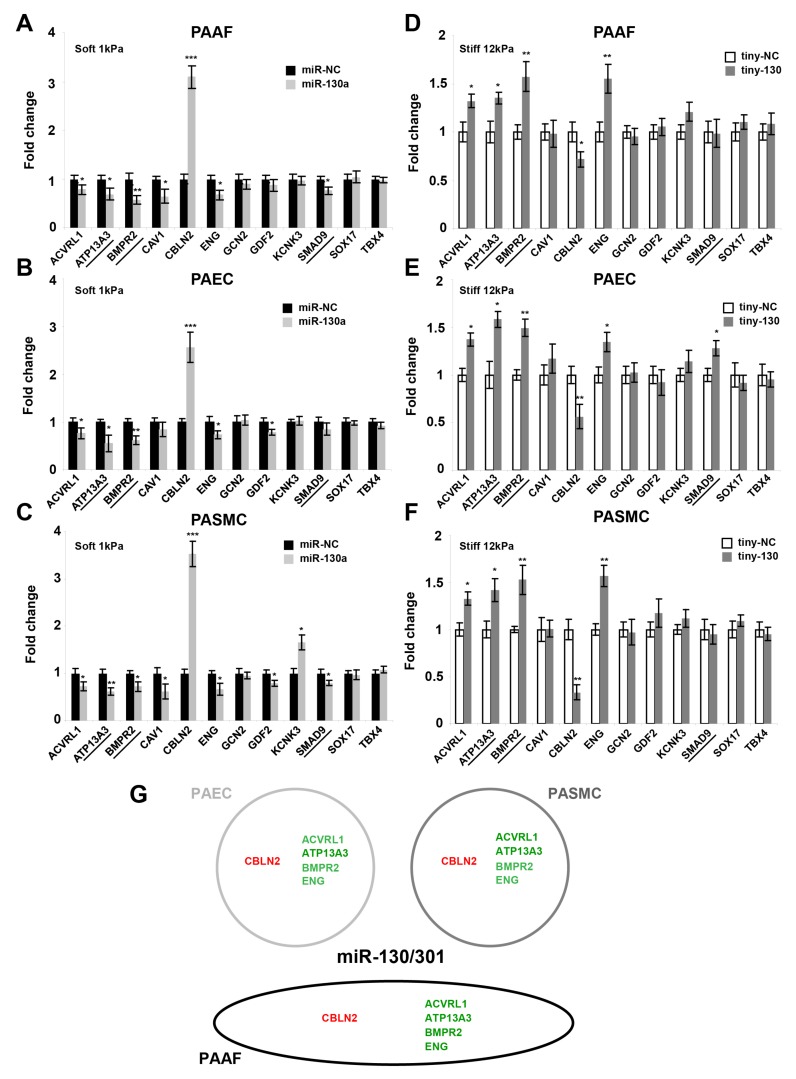
The miR-130/301 family modulates the expression of factors genetically associated with PAH. Following transfection with the indicated miR-130a mimic (**A**–**C**), miR-130/301 inhibitor (tiny-130) (**D**–**F**), or related scrambled controls, transcript levels of factors genetically associated with PAH were analyzed by RT-qPCR in PAAFs, PAECs, and PASMCs. Underlined genes: Predicted targets of miR-130/301 members (TargetScan). Mean expression in control groups (si-NC) was assigned a fold change of 1, to which relevant samples were compared. Data are expressed as mean ± SD (* *p* < 0.05, ** *p* < 0.01, *** *p* < 0.001) of three independent experiments. Paired samples were compared using a 2-tailed Student’s *t*-test. (**G**) Schematic of the main results. Red: upregulated genes. Green: downregulated genes, upon miR-130a forced expression.

MicroRNA-mediated feed-back and feed-forward loops are recurrent network motifs to enhance the robustness of gene regulation in mammalian genomes [20]. Thus, given the regulation of miR-130/301 by factors associated with hereditary PAH, we investigated whether the miR-130/301 family reciprocally controls the expression of these factors in PA vascular cells (Figure 3). First, we performed an in silico analysis of miR-130/301 target genes, using the well-validated microRNA target prediction algorithm Targetscan [21] and revealing canonical and conserved binding sites for miR-130/301 in the 3’ untranslated regions (3’UTR) of *ATP13A3*, *BMPR2*, and *SMAD9* transcripts. To mechanistically confirm these predictions, we performed gain-of-fuction experiments in vitro by forcing expression of miR-130a via transfection of oligonucleotide mimics in vascular cells cultivated in soft matrix (1 kPa). Across PAAFs (Figure 3A), PAECs (Figure 3B), and PASMCs (Figure 3C), such mimics decreased the transcript levels of *ATP13A3* and *BMPR2* as well as *ACVRL1* and *ENG*. Mimics also increased *CBLN2* transcript. Not all effects of miR-130a were consistent across cell types, as forced miR-130a expression decreased *CAV1* and *SMAD9* transcript levels only in PAAFs and PASMCs, decreased *GDF2* transcript only in PAECs and PASMCs, and increased expression of *KCNK3* solely in PASMCs. Loss-of-fuction experiments were also performed in cells cultivated in stiff matrix (12 kPa), whereby inhibition of the entire miR-130/301 family was achieved using a specifically designed short locked nucleic acid oligonucleotide (“tiny LNA”) with antisense complementarity only to the seed sequence of this microRNA family [18]. Converse to the effects of forcing miR-130a expression, inhibition of the miR-130/301 family in stiff matrix increased the transcript levels of *ACVRL1*, *ATP13A*, *BMPR2*, and *ENG* in PAAFs (Figure 3D), PAECs (Figure 3E), and PASMCs (Figure 3F), but decreased the expression of *CBLN2*.

Taken together, these results demonstrate the pervasive influence of miR-130/301 family in controlling factors genetically linked to PAH, indicating that the miR-130/301 family often is both necessary and sufficient to modulate some of these stiffness-dependent factors (Figure 3G).

### 2.4. Factors Genetically Associated with PAH Broadly Impact the PPARγ-ApoE-LRP8-Matrix Remodeling Axis

**Figure 4:**
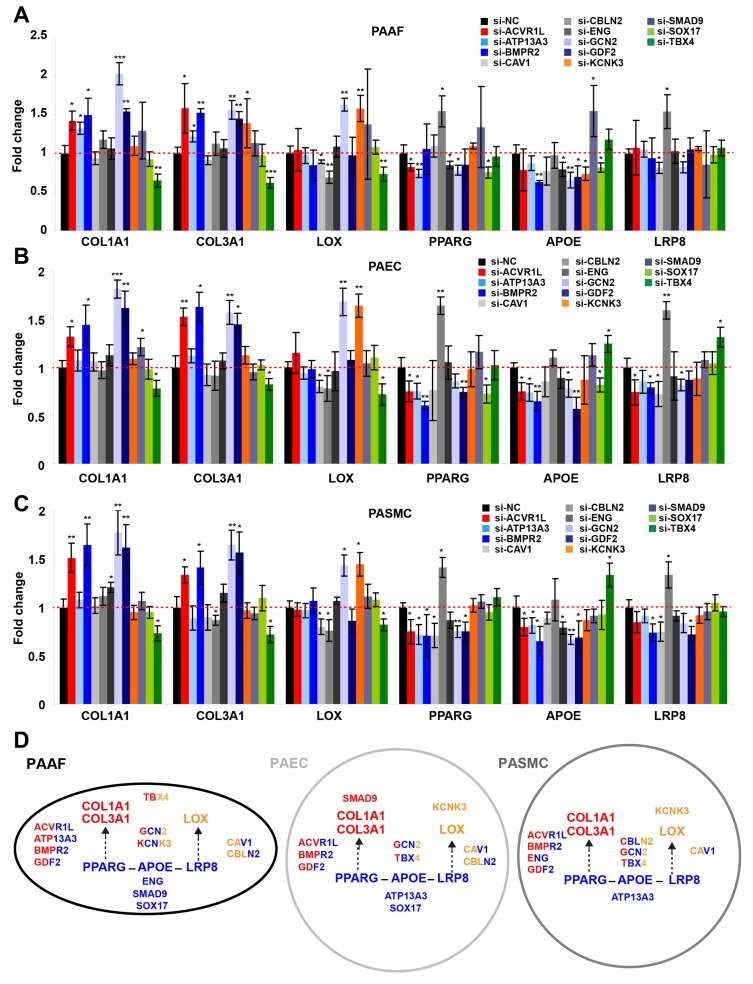
Upstream factors linked to hereditary PAH modulate the PPARγ-APOE-LRP8 axis, which in turn controls pulmonary vascular matrix stiffening. (**A**–**C**) Following transfection with the indicated siRNAs, transcripts related to the PPARγ-APOE-LRP8-matrix remodeling axis were analyzed by RT-qPCR in PAAFs (**A**), PAECs (**B**), and PASMCs (**C**). Mean expression of a given miRNA in the control group (si-NC) was assigned a fold change of 1, to which corresponding miRNA levels in experimental groups were compared. Data are expressed as mean ± SD (* *p* < 0.05, ** *p* < 0.01, *** *p* < 0.001) of three independent experiments. One-way ANOVA and post-hoc Tukey tests were used for group comparisons. (**D**) Schematic of the main results. In each cell type, the central downstream PPARγ-APOE-LRP8-LOX-collagen axis is drawn. Factors genetically linked to PAH are strategically placed next to the portion of the axis which they regulate; font is also color-coded based on these connections. Blue: genes related to the PPARγ-APOE-LRP8 axis; Red: genes related to fibrillar collagen; Orange: genes related to LOX.

Recently, we described the miR-130/301-PPARγ-APOE-LRP8-matrix remodeling loop as a central positive feedback mechanism in PAH and fibrotic diseases [10,22]. Given our current findings that indicate mutual and reciprocal regulation between miR-130/301 members and factors associated with hereditary PAH (Figure 2 and Figure 3), we investigated whether factors genetically linked to PAH interact with this central pathogenic axis (Figure 4). We found that siRNA-mediated knockdown of *KCNK3* increased LOX transcript expression in PAAFs (Figure 4A), PAECs (Figure 4B), and PASMCs (Figure 4C). Moreover, inhibition of *ACVRL1*, *ATP13A3*, *BMPR2*, *CAV1*, *CBLN2*, *ENG*, *GCN2*, *GDF2*, *TBX4*, and *SOX17* all differentially modulated the expression of the factors contributing to the central PPARγ-APOE-LRP8 axis. While siRNA-mediated knockdown of GCN2 and TBX4 modulated transcript expression of specific fibrillar collagen genes (*COL1A1* and *COL3A1*) and LOX, inhibition of others only modified a subset of these downstream matrix remodeling genes. Namely, inhibition of *ACVRL1*, *BMPR2*, and *GDF2* increased transcript expression of fibrillar collagen genes (*COL1A1* and *COL3A1*), while inhibition of *CAV1* and *CBLN2* decreased LOX.

These findings reveal that the network of factors linked to hereditary PAH all regulate the miR-130/301-PPARγ-LRP8 axis in various vascular cell types, thus controlling downstream collagen and collagen remodeling genes crucial to ECM stiffening (Figure 4D). As such, these findings define the unique central importance of this ECM-relevant molecular circuitry among seemingly disparate genetic factors in PAH, thus solidifying the principle of vascular stiffness as a primary driver of PAH.

### 2.5. A Computational Network Biology Approach Predicts Additional Molecular Pathways Linking Genes of Heritable PAH to the miR-130/301-ECM Axis

**Figure 5:**
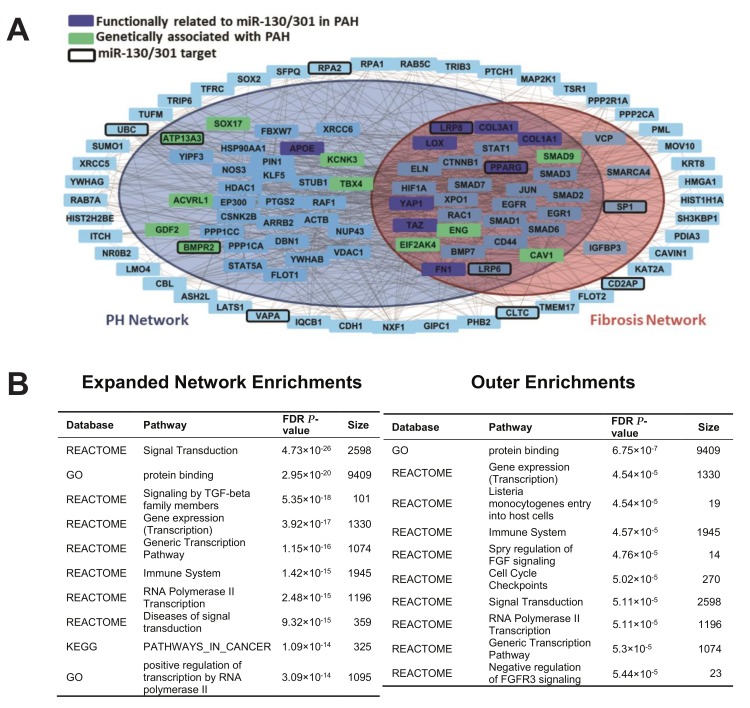
A network bioinformatics approach predicts the complex and overlapping relationships among factors genetically associated with PAH with known miR-130/301 target genes, PH genes, and fibrosis genes. (**A**) Network model. Gray lines indicate an interaction between two genes. Genes in dark blue are functionally related to miR-130/301 members in PAH, and genes in green are associated with heritable PAH. Genes with a thickened border are predicted targets of miR-130/301 in TargetScan [21]. Genes found in the larger blue circle are common to the PH Network, and those found in the orange circle are common to the Fibrosis Network. (**B**) In the left table, top-ranked pathways via gene set enrichment analysis (GSEA) are listed of genes found in the PH and Fibrosis Networks interacting with known miR-130/301 target genes and factors genetically associated with PAH (e.g., genes located in the inner colored circles of (**A**)). In the right table, top-ranked pathways are listed of genes interacting with miR-130/301 target genes and factors associated with heritable PAH found *outside* the PH/Fibrosis Networks (e.g., genes located outside the colored circles of (**A**)).

Based on the increasingly apparent convergent actions of factors important in hereditary PAH on miR-130/301 pathobiology and vascular stiffening, we endeavored to utilize a computational network biology approach to predict and visualize more comprehensively the functional interactions among these factors. To do so, we first constructed a PH gene network and fibrosis network, adapted from our prior published methodology [18]. In this case, for the PH network, we curated human genes known to play a role in PH from a literature search [18] and from the Pulmonary Arterial Hypertension Knowledge Base (PAHKB) [23]. We mapped functional interactions among these genes and with first-degree interactors via incorporating gene and protein interaction data from public databases, including DIP, BioGRID, Corum, InnateDB, IntAct, MINT, and MatrixDB [24,25,26,27,28,29,30]. A Fibrosis Network was similarly adapted from our prior report [22], with updated modifications including YAP/TAZ-dependent fibrotic factors in PAH [10]. Functional interactions of the known factors genetically associated with PAH and known target genes of miR-130/301 were then mapped into the PH and Fibrosis Networks (Figure 5A, Table S1). Importantly, and consistent with our experimental data (Figure 1, Figure 2, Figure 3 and Figure 4), a key overlap was observed among the PH and Fibrosis Networks, encompassing known miR-130/301 targets (*LRP6*, *LRP8*, *PPARγ*, and *SP1*), specific PAH hereditary factors (*ENG*, *CAV1*, *SMAP9*, and *EIF2AK4*), as well as multiple genes functionally related to miR-130/301 in PAH (*COL1A1*, *COL3A1*, *LOX*, *YAP1*, *TAZ*, and *FN1*). Importantly, while a number of these interactions were validated independently by our experimental studies (Figure 1, Figure 2, Figure 3 and Figure 4), this intersection also provided insight into numerous novel connections, thus offering a more complete functional roadmap for deciphering the relationships among these factors. Specifically, there were a number of genes implicated in these relationships that did not factor into either the pre-established PH or Fibrosis Networks (outer ring of genes, Figure 5A). While gene set enrichment analysis (GSEA) [31] of the PH and Fibrosis Networks predictably identified pathways involved in fibrosis and vascular stiffness (i.e., TGF-β/BMP signaling among others), GSEA of the non-PH/non-fibrosis genes indicated a number of novel pathways, such as immune activation, cell cycle checkpoint regulation, and DNA damage [32,33], never associated with miR-130/301, vascular stiffening, or several of these factors of hereditary PAH (Figure 5B, Tables S2 and S3). Thus, beyond the experimentally confirmed relationships defined in Figure 1, Figure 2, Figure 3 and Figure 4, computational network predictions implicate a range of novel functional connections and pathways mediating the association of the miR-130/301-ECM axis with the molecular origins of hereditary PAH.

## 3. Discussion

ECM stiffening, remodeling, and downstream mechanotransduction are recognized as causative drivers of multiple diseases, including PAH, by sustaining cell activities including proliferation, migration, apoptosis resistance, and metabolic rewiring [34]. Particularly in PAH, matrix remodeling and downstream vascular stiffening are increasingly appreciated as causative drivers of the disease process, promoted at least in part by the mechanosensitive miR-130/301 family and perivascular matrix remodeling and stiffening [10]. In this study, we found that seemingly disparate genetic drivers of PAH may activate this self-sustaining, positive feedback loop. These results are significant, as they emphasize the causative importance of this pathway in PAH pathogenesis and reinforce the attractive potential of developing tailored therapies for this molecular circuit of fibrosis. Moreover, our results indicate that, along with the TGF/BMP pathway, the miR-130/301-ECM axis serves as a central pathway where the functions of multiple factors of heritable PAH converge.

In light of the as-of-yet unexplained observation of incomplete genetic penetrance in heritable PAH, it has been proposed that the development of PH may originate from the interaction of a genetic state predisposed to disease along with one or more inciting stimuli (a “multiple-hit hypothesis”) [35]. More recently, PAH patients have been reported to carry multiple gene mutations in at least two distinct pathogenic loci [36,37], furthering the hypothesis that these multiple hits may originate from the complex genetic inheritance of several mutations. Our findings of convergent, miR-130/301-specific actions of seemingly disparate factors relevant to hereditary PAH now offer new insights into this multiple-hit hypothesis. Specifically, our findings could suggest that higher penetrance or more severe manifestation of disease may be observed in heritable PAH, particularly when multiple gene mutations converge upon the central miR-130/301-ECM axis. Alternatively, future work will be imperative to determine if other genomic polymorphisms associated with ECM biology are also linked to heritable PAH and could contribute to disease manifestation. In addition, considering that the miR-130/301 members are upregulated by other acquired triggers of PH such as stiffness, hypoxia, and inflammatory cytokines [10,18], our work suggests that a synergy of diverse disease exposures centrally regulating the miR-130/301-ECM remodeling axis may also be essential to the manifestation of both heritable and non-heritable forms of PAH alike.

Our findings also offer insight at the level of individual genes linked to heritable PAH, as their complete pathogenic actions have not been fully defined. This is particularly true for recently identified effectors *ATP13A3*, *CBLN2*, *GCN2*, *SOX17*, and *TBX4* [2,5,7]. Our data not only link their functions to miR-130/301 biology and matrix remodeling but also define a cell-type specificity among PAAFs, PAECs, and PASMCs in many of those activities. As specific examples, ATP13A3 is a poorly characterized P-type ATPase. Although the precise substrate specificity is unknown, ATP13A3 plays a role in polyamine transport [38]. Previously, it was shown that loss of ATP13A3 inhibits proliferation of endothelial cells and increases apoptosis in serum-deprived conditions [5]. Consistent with the previously reported role of miR-130/301 family on context-dependent endothelial apoptosis [18], and with the paradigm that endothelial apoptosis is a major trigger for the initiation of PAH [39], we now have demonstrated that depletion of ATP13A3 induces miR-130/301 expression and forced miR-130a expression reciprocally decreases ATP13A3 (Figure 3B,E), suggesting a positive feedback loop consistent with promoting endothelial cell apoptosis and PAH. *SOX17* encodes the SRY-box containing transcription factor 17, which plays a fundamental role in angiogenesis and arteriovenous differentiation [40,41]. Moreover, conditional deletion of SOX17 in mesenchymal progenitors has been reported to impair formation of lung microvessels [42]. Adding to these insights, we observed that a *SOX17*-ECM remodeling-miR-130/301 axis is active in both PAAFs and PAECs but not in PASMCs (Figure 1 and Figure 4). *CBLN2* belongs to the cerebellin gene family, a group of secreted neuronal glycoproteins (Cbln1-4) [43]. CBLN2 has been previously reported to be expressed mainly in the brain but has also be found in PAH lung vasculature, particularly in diseased PAECs [9]. Consistent with these observations, we found that both matrix stiffening (Figure 1) and forced miR-130a expression induced CBLN2 expression (Figure 3). In turn, inhibition of CBLN2 activated the expression of the PPARγ-APOE-LRP8 gene axis (Figure 4). Together, these results suggest that CBLN2 activation induces the miR-130/301-ECM remodeling feedback loop. Finally, *TBX4* encodes the early embryonic transcription factor T-box gene 4. T-box genes are transcription factors known to be involved in several developmental and cardiovascular diseases [44]. Recently, TBX4 was found to play an important role in lung fibrosis in mice via its actions in αSMA+ myofibroblasts and COL1α1+ fibroblasts [45]. Consistent with its role in fibrosis, we found that TBX4 knockdown decreased *COL1A1*, *COL3A1*, and *LOX* expression (Figure 4). While these results may seem contradictory with the fact that missense mutations (e.g., loss-of-function) in *TBX* are associated with PAH, it is important to note that TBX4 mutations are associated with childhood-onset PAH, but the prevalence of PAH in adults with TBX4 mutation carriers is low [7]. Given the role of TBX4 during embryonic development and airway branching, it is tempting to speculate that while germline mutations of TBX4 increase the prevalence of PAH, somatic mutation of TBX4 could slow PAH progression by blunting ECM remodeling. Clearly, future work is necessary to delve more completely into these new mechanistic connections, and by limiting our analyses to known members of these previously identified pathways, we acknowledge a certain level of confirmation bias to this study. Nonetheless, our data offer a much-needed roadmap for delineating the miR-130/301-dependent overlap of these individual genes associated with heritable PAH.

While our findings indicate a new set of functional connections among the genes of heritable PAH with miR-130/301 and vascular stiffness, there are a number of concepts relevant to fundamental pathogenesis and applied therapeutic development that await exploration. First, our study utilized artificial means of forcing and inhibiting gene expression, and our readouts were simple quantification of gene transcript levels. Notably, the majority of genetic mutations reported to be associated with PAH are missense single nucleotide mutations, frameshift mutations (deletions or insertions), and nonsense mutations (stop codon gains), suggesting that these are associated with loss-of-function [2]. However, siRNA knockdown may not fully recapitulate the molecular underpinning of these genetic mutations, particularly if the relationship of these genes to miR-130/301 and matrix remodeling depend on precise gene “dosage.” As such, future work will be necessary to study the functional impact of individual mutations and polymorphisms linked to PAH —potentially through use of inducible pluripotent stem cell technology and/or CRISPR/Cas9 technology [46]—and how the specific levels of altered expression that result may interact with the miR-130/301-ECM network. Second, our study only focused on pulmonary vascular cell types. There exists an emerging notion that several extrapulmonary organs (i.e., heart, skeletal muscle, gut, and adipose tissue, among others) demonstrate vascular and metabolic abnormalities in PAH, suggesting a systemic, rather than exclusively pulmonary vascular, disease [47,48,49,50]. Failure of the right ventricle (RV) in PAH has been particularly linked to fibrosis. Furthermore, PPARγ activation has been shown to reverse these epigenetic, transcriptional, and metabolic alterations in the RV and prevent PAH development in rodent models of PAH [51]. As such, it remains an intriguing question whether factors associated with heritable PAH can also activate this miR-130/301-dependent positive feedback loop in the RV and whether targeted therapy that modulates this axis should also directly address RV failure. Finally, given the striking convergence of actions of multiple genes implicated in heritable PAH, there is a compelling possibility of synergistic therapeutic effects of a rational combination of pharmacologic PPARγ activation along with the unique application of shortmer technology to repress the miR-130/301 family. However, due to the varying levels of cell-type specificity and gene function overlap defined in our study, we also see an increasing need to predict and more precisely target such therapies to the target site(s) and molecular circuitry of interest. Consequently, we envision that further experimental validation of PAH network architecture, as led by computational prediction strategies (such as in Figure 5), may have a substantial impact on such systems pharmacology methods in PAH in the future.

In sum, we report that a network of upstream factors genetically linked to PAH converges upon the miR-130/301-PPARγ-LRP8 axis with implications for controlling matrix remodeling and pulmonary vascular disease manifestation. While these results await validation in vivo, these functional relationships emphasize the causative importance of matrix and vascular stiffening in PAH and suggest a set of new and even surprising implications for how heritability and genetic penetrance in PAH may manifest over time.

## 4. Materials and Methods

### 4.1. Cell Culture and Cell Culture Reagents

Primary human pulmonary arterial endothelial cells (PAECs) were grown in EGM-2 cell culture media (Lonza, Basel, Switzerland), and experiments were performed at passages 3 to 6. Primary human pulmonary arterial smooth muscle cells (PASMCs) were cultured in SmGM-2 cell culture media (Lonza), and experiments were performed at passages 3 to 9. Primary human pulmonary arterial adventitial fibroblast cells (PAAFs) were purchased (Sciencell Research Laboratories, Carlsbad, CA, USA) and grown in FGM cell culture media (Lonza). Experiments were performed at passages 3 to 6. At baseline, cultured cells were grown in collagen-coated plastic (50 μg/mL) at 37 °C in a humidified 5% CO_2_ atmosphere. Collagen-coated hydrogel was purchased from Matrigen (Brea, CA, USA).

### 4.2. Oligonucleotides and Transfection

Pre-miRNA oligonucleotides (pre-miR-130a, negative control pre-miR-NC1, and premiR-NC2) and custom-designed tiny LNA oligonucleotides (tiny-130: 5′-ATTGCACT-3′ and tiny-NC: 5′-TCATACTA-3′) were purchased from Thermo Fisher Scientific (Waltham, MA, USA) and Exiqon (Qiagen, Germantown, MD, USA), respectively. PAAFs, PAECs, and PASMCs were plated in collagen-coated plastic (50 μg /mL) and transfected 24 h later at 70–80% confluence using pre-miRNA (5 nM), tiny-LNA (20 nM), or siRNA (25 nM) and Lipofectamine 2000 reagent (Thermo Fisher Scientific), according to the manufacturers’ instructions. Eight hours after transfection, cells were trypsinized and re-plated on hydrogel.

### 4.3. Messenger RNA and miRNA Extraction

Cells were homogenized in 1 ml of QiaZol reagent (Qiagen, Germantown, MD, USA). Total RNA content, including small RNAs, was extracted using the miRNeasy kit (Qiagen) according to the manufacturer’s instructions. Total RNA concentration was determined using a ND-1000 micro-spectrophotometer (NanoDrop Technologies, Wilmington, DE, USA).

### 4.4. Quantitative RT-PCR of Mature miRNAs

As we described previously [19], mature miRNA expression was evaluated using TaqMan MicroRNA Assays (Thermo Fisher Scientific) and the Applied Biosystems 7900HT Fast Real Time PCR device (Thermo Fisher Scientific). Expression levels were normalized to RNU48 and calculated using the comparative Ct method (2^−ΔΔ*C*t^). In order to ensure biological relevance, only Ct values <35 were utilized in the analysis.

### 4.5. Quantitative RT-PCR of Messenger RNAs

Messenger RNAs were reverse transcribed using the Multiscript RT kit (Thermo Fisher Scientific) to generate cDNA. cDNA was amplified via fluorescently labeled Taqman primer sets using an Applied Biosystems 7900HT Fast Real Time PCR device (Thermo Fisher Scientific). Fold-change of RNA species was calculated using the formula (2^−ΔΔ*C*t^), normalized to RPLP0 expression. In order to ensure biological relevance, only *C*t values <35 were utilized in the analysis.

### 4.6. Computational Gene Network Analysis

A network analysis was devised to visualize functional interactions among factors genetically associated with PAH and known miR-130/301 target genes, PH genes, and fibrosis genes. First, a consolidated interactome (CI) was constructed using gene and protein interaction data from public databases, including DIP, BioGRID, Corum, InnateDB, IntAct, MINT, and MatrixDB [24,25,26,27,28,29,30]. Second, we identified PH-relevant genes in the CI by finding genes (nodes) common to a defined PH gene network. To define this PH Network, we curated a list of genes known to play a role in pulmonary hypertension from a literature search [18] and from the Pulmonary Arterial Hypertension Knowledge Base (PAHKB) [23]. These seed genes were mapped onto the CI, and the largest connected component (LCC) was identified. Additional first-degree interactors from the interactome were iteratively incorporated into the LCC that would include otherwise disconnected PH seed genes. This expansion led to the PH network and consisted of 3139 interactions among 833 genes, 370 of which were common to the literature search and PAHKB. Third, to identify fibrosis-relevant genes, we utilized a previously constructed Fibrosis Network (1459 interactions among 350 genes) [22], with updated modifications via inclusion of YAP/TAZ-dependent fibrotic factors in PAH, as we reported [10]. With these available networks (the CI, PH Network, and Fibrosis Network), functional interactions of the factors associated with heritable PAH and target genes of miR-130/301 were then mapped into the PH and Fibrosis networks. Specifically, we sought to identify the largest connected component (LCC) connecting genes associated with heritable PAH and miR-130/301 gene targets. Additional first-degree interactors from the CI were iteratively incorporated into this LCC that would include otherwise disconnected genes of heritable PAH and miR-130/301 target genes. The final LCC included 109 genes and 694 interactions.

Two rounds of gene set enrichment analysis (GSEA) [31] were performed on this LCC. The first analysis included all 109 genes in the expanded network. The second included only the 45 genes not common to either the PH Network or Fibrosis Network. Annotations were acquired from The Gene Ontology [52,53], KEGG [54], REACTOME [55,56], and BioCarta [57].

### 4.7. Statistical Analysis

Cell culture experiments were performed at least three times. Each independent experiment was performed in triplicate, results of which were averaged for further statistical analysis. Paired samples were compared using a 2-tailed Student’s *t*-test for normally distributed data, while Mann-Whitney U non-parametric testing was used for non-normally distributed data. For comparisons among groups, one-way ANOVA and post-hoc Tukey testing was performed. A P-value less than 0.05 was considered significant. No samples were excluded.

## Supplementary Materials

Supplementary materials can be found at http://www.mdpi.com/1422-0067/19/8/2289/s1.
