# Supplementary material for: Factors Associated with Heritable Pulmonary Arterial Hypertension Exert Convergent Actions on the miR-130/301-Vascular Matrix Feedback Loop
Source: Int J Mol Sci. 2018 Aug 4;19(8):2289. doi: 10.3390/ijms19082289 (PMC6121519; doi:10.3390/ijms19082289)
Supplement: Supplementary file 1 [file ijms-19-02289-s001.pdf]

Supplementary Material

Factors associated with heritable pulmonary arterial hypertension exert convergent actions on the miR-130/301-vascular matrix feedback loop

Thomas Bertero, Adam L. Handen, and Stephen Y. Chan

Table S1: A catalog of each gene included in the network model along with the interacting network partners.

Table S2: Gene set enrichment analyses of genes within the PH and Fibrosis Networks interacting with miR-130/301 target genes and factors associated with heritable PAH.

Table S3: Gene set enrichment analyses of genes outside of the PH and Fibrosis Networks interacting with miR-130/301 target genes and factors associated with heritable PAH.

Figure S1: Efficiency of gene knockdown by inhibitory RNA in pulmonary vascular cell types.

Table S1: A catalog of each gene included in the network model along with the interacting network partners.

Gene	Interacting Partners
PTGS2	HDAC1, VCP, FN1, SUMO1, EGFR, JUN, CTNNB1, CLTC, VDAC1, EP300, CAV1
MOV10	XRCC6, RAB5C, HIST1H1A, CAV1, RPA1, LRP8, LMO4, TAZ, RPA2, CTNNB1, STUB1, SFPQ, UBC, TRIP6, DBN1, VDAC1, FBXW7, RAF1, SH3KBP1, YAP1
SUMO1	PTGS2, JUN, PML, VCP, PPARG, KLF5, UBC, SOX2, YAP1
IQCB1	XPO1, RAC1, VCP, YWHAB, HSP90AA1, ACTB, KRT8, RAF1, LOX
DBN1	CAVIN1, MOV10, PPP1CC, FN1, PPP1CA, CD44, CLTC, ARRB2, FLOT1, FLOT2, SP1, CDH1, ACTB, CAV1
KCNK3	YWHAB, EGR1
CAV1	PPP2R1A, CAVIN1, MOV10, ITCH, PTGS2, EGFR, VAPA, CTNNB1, LATS1, NOS3, CLTC, DBN1, VDAC1, KRT8, TMEM17, TFRC, CDH1, RAB7A, ACVRL1, NXF1, RAB5C, RAC1, CD44, PDIA3, VCP, PPARG, SMAD2, YIPF3, LRP6, PPP1CA, PPP2CA, RPA1, RPA2, BMPR2, FLOT1, FLOT2, UBC, HSP90AA1, ACTB, PTCH1
STUB1	PPP2R1A, MOV10, NXF1, EGFR, SMAD1, HIF1A, SMAD3, VCP, PPARG, ARRB2, SMAD2, UBC, HSP90AA1, RAF1, YAP1
NR0B2	HDAC1, PPARG, FN1
TAZ	BMP7, MOV10, LATS1, LRP6, NXF1
CDH1	HDAC1, SMAD7, EGFR, VAPA, CTNNB1, DBN1, KRT8, XRCC5, CAV1, STAT1, YWHAB, SH3KBP1, PPARG, GIPC1, EP300, YAP1, PPP1CA, CBL, CD2AP, BMPR2, FLOT1, UBC, TRIP6, HSP90AA1
RAB5C	RAB7A, MOV10, NXF1, FN1, PHB2, RPA1, CAVIN1, VAPA, RPA2, TFRC, ARRB2, FLOT1, FLOT2, VDAC1, SMARCA4, CAV1
SP1	HDAC1, COL1A1, NXF1, IGFBP3, SMAD3, JUN, HIF1A, HMGA1, CTNNB1, PPARG, SFPQ, COL3A1, EGR1, DBN1, PML, HSP90AA1, PIN1, EP300, SMARCA4, SMAD2
VAPA	CAVIN1, RAB5C, FN1, PHB2, EGFR, VCP, FLOT1, FLOT2, CDH1, VDAC1, SMARCA4, CAV1
GIPC1	CDH1, LRP8, ENG
BMP7	SMAD3, BMPR2, SMAD1, TAZ, ENG
FN1	HDAC1, PPP2R1A, PTGS2, PHB2, EGFR, HIF1A, CLTC, TRIB3, DBN1, KRT8, XRCC5, XRCC6, XPO1, NR0B2, TFRC, YWHAB, YWHAG, RAB7A, ELN, RAB5C, RAC1, CD44, HMGA1, VCP, IGFBP3, SFPQ, VAPA, PPP1CA, PPP2CA, RPA1, RPA2, STAT5A, FLOT1, FLOT2, UBC, HSP90AA1, ACTB, CSNK2B, TUFM
PPP2CA	PPP2R1A, FN1, RAC1, EGR1, RPA1, RPA2, VCP, PIN1, EP300, RAF1, CSNK2B, CAV1
SH3KBP1	MOV10, NXF1, EGFR, RPA1, CBL, CD2AP, CLTC, UBC, CDH1, LOX, RAF1
BMPR2	BMP7, CD44, PPP1CC, TSR1, GDF2, SMAD6, SMAD7, TRIB3, CDH1, CAV1
UBC	PPP2R1A, CAVIN1, MOV10, ITCH, SUMO1, EGFR, SMAD1, ARRB2, HIST2H2BE, VDAC1, CD2AP, CAV1, CDH1, STUB1, JUN, NUP43, YWHAB, SH3KBP1, NXF1, RAC1, YWHAG, VCP, PPARG, SFPQ, EP300, FN1, RPA1, CBL, RPA2, FLOT1, FLOT2, COL1A1, TUFM
TRIP6	XPO1, MOV10, ENG, CDH1, PIN1, YAP1

ACTB	VCP, NOS3, SMAD9, PPP1CC, FN1, PPP1CA, EGFR, IQCB1, RPA1, CBL, SMAD3, CTNNB1, ARRB2, RAC1, RPA2, DBN1, EP300, SMARCA4, CSNK2B, CAV1
CAVIN1	RAB7A, RAB5C, JUN, EGFR, VAPA, FLOT2, UBC, DBN1, YAP1, CAV1
HIF1A	HDAC1, VCP, FN1, ENG, STUB1, SP1, EGR1, JUN, SMAD3, CTNNB1, HSP90AA1, EP300, SMARCA4, FBXW7
EIF2AK4	ARRB2, PPP1CC, PPP1CA
CLTC	PPP2R1A, XRCC6, PPP1CC, FN1, PPP1CA, PHB2, RAC1, RPA1, PTGS2, RPA2, NXF1, ARRB2, DBN1, VDAC1, EP300, SH3KBP1, TUFM, CAV1
TRIB3	BMPR2, CSNK2B, FN1, SMAD3
COL3A1	SP1
CD2AP	EGFR, CBL, UBC, CDH1, LOX, SH3KBP1
XRCC5	XRCC6, FN1, CDH1, RPA1, EGFR, CBL, RPA2, PPARG, ARRB2, KAT2A, SOX2, VDAC1, EP300, YAP1
XRCC6	MOV10, NXF1, FN1, PDIA3, SMAD7, RPA1, KAT2A, HMGA1, SMAD3, PPARG, ARRB2, CLTC, RPA2, VDAC1, EP300, XRCC5, PIN1, YAP1
XPO1	ASH2L, NXF1, STAT1, FN1, PPP1CA, TRIP6, IQCB1, EGFR, SMAD1, CBL, CSNK2B, CTNNB1, PPARG, KLF5, ARRB2, SOX2, HSP90AA1, RAF1, YWHAG, TUFM, YAP1
APOE	LRP8, NOS3, VDAC1
EGR1	HDAC1, MAP2K1, ENG, PPP2CA, JUN, SMAD7, EGFR, SMAD1, HIF1A, KCNK3, CTNNB1, SMAD3, KLF5, CBL, SP1, CD44, EP300, YAP1
JUN	HDAC1, PTGS2, CAVIN1, ITCH, SUMO1, HIF1A, SMAD3, CTNNB1, PIN1, STAT1, EGR1, KLF5, PML, FBXW7, SP1, HMGA1, PPARG, SMAD2, EP300, YAP1, UBC, HSP90AA1
SOX17	CTNNB1
TFRC	RAB7A, TMEM17, FN1, RPA1, EGFR, RPA2, RAB5C, CAV1
RAB7A	PPP2R1A, CAVIN1, TMEM17, FN1, PHB2, RPA1, RPA2, VCP, RAB5C, TFRC, FLOT1, FLOT2, VDAC1, CAV1
ACVRL1	HSP90AA1, ENG, SMAD1, CSNK2B, CAV1
NXF1	XPO1, XRCC6, RAB5C, STUB1, SP1, LRP8, LMO4, SFPQ, TAZ, CSNK2B, CTNNB1, YAP1, CLTC, UBC, HSP90AA1, VDAC1, CAV1, RAF1, SH3KBP1, FBXW7
ENG	BMP7, ACVRL1, EGR1, HIF1A, ARRB2, TRIP6, GIPC1, TUFM
PPARG	HDAC1, ASH2L, PHB2, EGFR, KAT2A, SMAD3, CTNNB1, MAP2K1, PIN1, SMAD2, XRCC5, CAV1, XPO1, STAT1, CDH1, STUB1, SMARCA4, JUN, SUMO1, KLF5, YWHAB, PML, XRCC6, YWHAG, SP1, HMGA1, IGFBP3, SFPQ, NR0B2, EP300, LMO4, STAT5A, UBC
SFPQ	HDAC1, MOV10, UBC, FN1, PPP1CA, EGFR, RAC1, RPA1, RPA2, PPARG, ARRB2, SP1, PIN1, NXF1
YIPF3	ATP13A3, CAV1
IGFBP3	FN1, CD44, HMGA1, PPARG, SP1, COL1A1
CBL	XPO1, EGR1, ITCH, SMAD7, EGFR, SMAD3, CTNNB1, CDH1, STAT5A, FLOT1, UBC, YWHAB, ACTB, CD2AP, XRCC5, SH3KBP1, LOX
FLOT1	RAB7A, RAB5C, FN1, CBL, VAPA, FLOT2, UBC, CDH1, DBN1, CAV1

FLOT2	RAB7A, TMEM17, RAB5C, FN1, CD44, EGFR, CAVIN1, VAPA, FLOT1, UBC, DBN1, VDAC1, CAV1
HDAC1	PPP2R1A, PTGS2, PHB2, SMAD7, EGFR, SMAD1, HIF1A, SMAD3, CTNNB1, SOX2, PIN1, PML, SMARCA4, STAT1, NR0B2, EGR1, JUN, KLF5, CDH1, SP1, PPARG, SFPQ, SMAD2, EP300, YAP1, PPP1CC, FN1, PPP1CA, LMO4, STAT5A, CSNK2B
SMAD9	ASH2L, SMAD7, LMO4, KAT2A, SMAD3, SMAD2, ACTB
ASH2L	XPO1, PPP1CC, CTNNB1, PPARG, EP300, SMAD9
SMAD6	ITCH, SMAD7, SMAD1, SMAD2, BMPR2, YAP1
SMAD7	HDAC1, SMAD9, ITCH, SMAD6, EGR1, XRCC6, SMAD1, CBL, SMAD3, CTNNB1, BMPR2, SMAD2, CDH1, EP300, YAP1
SMAD1	BMP7, XPO1, ACVRL1, STUB1, SMAD6, SMAD7, KAT2A, SMAD3, SMAD2, EGR1, UBC, EP300, HDAC1, YAP1
SMAD2	HDAC1, PPP2R1A, SMAD9, ITCH, SMAD6, SMAD7, SMAD1, KAT2A, SMAD3, CTNNB1, PIN1, SMARCA4, CAV1, STUB1, JUN, KLF5, PML, SP1, PPARG, EP300, YAP1, COL1A1
SMAD3	HDAC1, PPP2R1A, SMAD9, ITCH, SMAD7, SMAD1, KAT2A, CTNNB1, TRIB3, PIN1, SMAD2, SMARCA4, XRCC6, STUB1, EGR1, JUN, KLF5, PML, SP1, PPARG, HIF1A, EP300, YAP1, BMP7, RPA1, CBL, COL1A1, ACTB
ARRB2	PPP2R1A, ITCH, EGFR, MAP2K1, CLTC, DBN1, XRCC5, XRCC6, XPO1, STAT1, STUB1, RAF1, YWHAB, YWHAG, RAB5C, ENG, VCP, SFPQ, HIST1H1A, RPA1, EIF2AK4, UBC, ACTB
TBX4	NUP43, FBXW7
VDAC1	PTGS2, RAB7A, NOS3, MOV10, CLTC, RAB5C, PHB2, RAF1, VAPA, NXF1, APOE, FLOT2, UBC, XRCC6, XRCC5, CSNK2B, CAV1
KRT8	CDH1, IQCB1, FN1, CAV1
RAF1	XPO1, PPP2R1A, MOV10, PPP1CC, PPP2CA, IQCB1, EGFR, MAP2K1, SH3KBP1, VCP, STUB1, LATS1, ARRB2, YWHAB, HSP90AA1, LOX, VDAC1, YWHAG, PIN1, NXF1
TMEM17	RAB7A, LRP6, LRP8, TFRC, FLOT2, CAV1
STAT1	XPO1, HDAC1, CDH1, JUN, EGFR, VCP, PPARG, STAT5A, ARRB2, PML, EP300, SMARCA4, YAP1
PML	HDAC1, STAT1, SUMO1, JUN, EGFR, SMAD2, SMAD3, CTNNB1, PPARG, SP1, PIN1, SMARCA4, CSNK2B, YAP1
FBXW7	MOV10, NXF1, JUN, HIF1A, CTNNB1, KLF5, TBX4, HSP90AA1, PIN1, YAP1
ELN	LOX, FN1
HMGA1	FN1, IGFBP3, EGFR, JUN, PPARG, SP1, HSP90AA1, XRCC6
KAT2A	SMAD9, SMAD1, SMAD2, SMAD3, CTNNB1, PPARG, HIST2H2BE, XRCC5, XRCC6
EP300	HDAC1, PTGS2, CLTC, ASH2L, SMAD7, SMAD1, SMAD2, SMAD3, CTNNB1, HIST2H2BE, PIN1, XRCC5, XRCC6, STAT1, EGR1, JUN, KLF5, SP1, CDH1, CD44, PPARG, HIF1A, HIST1H1A, YAP1, PPP2CA, STAT5A, UBC, ACTB
HIST1H1A	MOV10, LOX, EP300, ARRB2
YAP1	HDAC1, PPP2R1A, CAVIN1, MOV10, ITCH, SUMO1, SMAD6, SMAD7, EGFR, SMAD1, SMAD2, SMAD3, CTNNB1, LATS1, PIN1, SMARCA4, XRCC6, XPO1, STAT1, TSR1, XRCC5, EGR1, JUN, PHB2, KLF5, YWHAB, CDH1, YWHAG, FBXW7, NXF1, PDIA3, VCP, PML, EP300, PPP1CA, MAP2K1, STUB1, TRIP6, PTCH1

LRP6	CTNNB1, TMEM17, TAZ, CAV1
LRP8	APOE, MOV10, TMEM17, EGFR, GIPC1, NXF1
LMO4	HDAC1, MOV10, NXF1, PPARG, CSNK2B, SMAD9
MAP2K1	EGR1, VCP, PPARG, ARRB2, YWHAB, RAF1, YAP1
STAT5A	HDAC1, STAT1, FN1, EGFR, CBL, PPARG, EP300, SMARCA4, CSNK2B
COL1A1	SP1, EGFR, SMAD2, SMAD3, IGFBP3, UBC
HSP90AA1	XPO1, PPP2R1A, NOS3, NXF1, FN1, STUB1, EGFR, IQCB1, JUN, HIF1A, HMGA1, VCP, ACVRL1, SP1, CDH1, CAV1, RAF1, TUFM, FBXW7
CSNK2B	HDAC1, PPP2R1A, ACVRL1, NXF1, FN1, PPP1CA, PPP2CA, LMO4, XPO1, RPA1, VCP, STAT5A, TRIB3, PML, ACTB, VDAC1, PPP1CC
PPP2R1A	HDAC1, RAB7A, FN1, PPP2CA, EGFR, SMAD2, SMAD3, VCP, STUB1, ARRB2, CLTC, UBC, HSP90AA1, YAP1, RAF1, CSNK2B, CAV1
ITCH	PTCH1, JUN, SMAD6, SMAD7, EGFR, CBL, SMAD3, LATS1, ARRB2, SMAD2, UBC, YAP1, CAV1
PHB2	HDAC1, RAB7A, RAB5C, FN1, VAPA, CTNNB1, PPARG, CLTC, VDAC1, TUFM, YAP1
EGFR	HDAC1, PPP2R1A, CAVIN1, ITCH, PTGS2, VAPA, PML, CTNNB1, ARRB2, CD2AP, XRCC5, CAV1, XPO1, STAT1, CDH1, STUB1, RAF1, EGR1, TFRC, YWHAB, SH3KBP1, CD44, COL1A1, PDIA3, HMGA1, PPARG, SFPQ, YAP1, FN1, LRP8, RPA1, CBL, RPA2, STAT5A, FLOT2, UBC, HSP90AA1, ACTB, PTCH1, TUFM
CTNNB1	HDAC1, PTGS2, MOV10, ASH2L, PHB2, SMAD7, EGFR, HIF1A, PML, SOX2, PIN1, SMAD2, SMARCA4, CAV1, XPO1, EGR1, JUN, SOX17, KLF5, CDH1, FBXW7, NXF1, SP1, SMAD3, PPARG, KAT2A, EP300, YAP1, LRP6, CBL, ACTB
LATS1	PPP1CC, ITCH, RAC1, PPP1CA, TAZ, YAP1, RAF1, CAV1
HIST2H2BE	LOX, EP300, SMARCA4, KAT2A, UBC
SOX2	HDAC1, XPO1, SUMO1, CTNNB1, LOX, XRCC5
LOX	ELN, HIST1H1A, IQCB1, CBL, CD2AP, HIST2H2BE, SOX2, RAF1, SH3KBP1
SMARCA4	HDAC1, STAT1, RAB5C, SP1, HIF1A, SMAD3, CTNNB1, PPARG, STAT5A, VAPA, HIST2H2BE, PML, ACTB, SMAD2, YAP1
PIN1	HDAC1, XRCC6, PPP2CA, SP1, JUN, TRIP6, SMAD3, CTNNB1, PPARG, SFPQ, SMAD2, PML, YAP1, EP300, RAF1, FBXW7
TSR1	BMPR2, RPA2, RPA1, YAP1
GDF2	BMPR2
ATP13A3	YIPF3
NUP43	TBX4, UBC
KLF5	XPO1, HDAC1, SUMO1, EGR1, JUN, SMAD2, SMAD3, CTNNB1, PPARG, YAP1, EP300, FBXW7
YWHAB	FN1, IQCB1, EGFR, CBL, KCNK3, VCP, PPARG, MAP2K1, ARRB2, UBC, CDH1, RAF1, YWHAG, YAP1
YWHAG	XPO1, FN1, VCP, PPARG, ARRB2, UBC, YWHAB, RAF1, YAP1
NOS3	HSP90AA1, ACTB, APOE, VDAC1, CAV1
RAC1	FN1, PPP2CA, IQCB1, LATS1, SFPQ, CLTC, UBC, ACTB, CAV1
CD44	FN1, EGR1, EGFR, IGFBP3, BMPR2, FLOT2, DBN1, EP300, CAV1

PDIA3	XRCC6, CAV1, EGFR, YAP1
VCP	PPP2R1A, PTGS2, SUMO1, IQCB1, HIF1A, MAP2K1, ARRB2, RAF1, CAV1, STAT1, STUB1, YWHAB, YWHAG, RAB7A, VAPA, YAP1, PPP1CC, FN1, PPP2CA, RPA2, UBC, HSP90AA1, ACTB, CSNK2B
PPP1CC	HDAC1, PPP1CA, ASH2L, RPA1, EIF2AK4, RPA2, VCP, LATS1, BMPR2, CLTC, DBN1, ACTB, RAF1, CSNK2B
PPP1CA	HDAC1, XPO1, PPP1CC, FN1, EIF2AK4, SFPQ, CLTC, CDH1, DBN1, ACTB, YAP1, CSNK2B, LATS1, CAV1
RPA1	RAB7A, MOV10, PPP1CC, FN1, XRCC6, PPP2CA, EGFR, TSR1, CSNK2B, SMAD3, RAB5C, SFPQ, TFRC, ARRB2, CLTC, UBC, ACTB, RPA2, XRCC5, SH3KBP1, CAV1
RPA2	RAB7A, MOV10, PPP1CC, FN1, PPP2CA, EGFR, RPA1, TSR1, VCP, RAB5C, SFPQ, TFRC, CLTC, UBC, ACTB, XRCC6, XRCC5, CAV1
PTCH1	CAV1, EGFR, ITCH, YAP1
TUFM	XPO1, FN1, ENG, PHB2, EGFR, CLTC, UBC, HSP90AA1

Table S2: Gene set enrichment analyses of genes within the PH and Fibrosis Networks interacting with miR-130/301 target genes and factors associated with heritable PAH. Pathways are listed in order of lowest to highest FDR p-value (Q).

Source	Annotation	Size	P	Q	Genes
GO	protein binding	9409	3.78E-11	6.75E-07	KRT8 RPA1 CLTC VAPA HMGA1 PML SFPQ TUFM IQCB1 PPP2R1A RPA2 PTCH1 GIPC1 CD2AP FLOT2 LATS1 ASH2L CDH1 PPP2CA HIST1H1A TRIP6 CBL TFRC TRIB3 RAB5C SH3KBP1 CAVIN1 NR0B2 KAT2A SUMO1 NXF1 RAB7A ITCH PDIA3 LMO4 MAP2K1 PHB2 MOV10 SOX2 XRCC5 UBC YWHAG
REACTOME	Gene expression (Transcription)	1330	1.05E-07	4.54E-05	RPA2 PPP2CA RPA1 UBC HIST2H2BE CAVIN1 PML MOV10 NR0B2 ASH2L ITCH PPP2R1A KAT2A SUMO1 YWHAG
REACTOME	Listeria monocytogenes entry into host cells	19	8.45E-08	4.54E-05	CBL SH3KBP1 CDH1 UBC
REACTOME	Immune System	1945	7.35E-08	4.57E-05	PDIA3 PPP2CA MAP2K1 SH3KBP1 CBL CLTC VAPA TRIB3 RAB5C PML XRCC5 RAB7A UBC ITCH PPP2R1A SOX2 SUMO1 CDH1
REACTOME	Spry regulation of FGF signaling	14	2.2E-08	4.76E-05	PPP2R1A PPP2CA CBL UBC
REACTOME	Cell Cycle Checkpoints	270	1.39E-07	5.02E-05	RPA2 PPP2CA RPA1 HIST2H2BE UBC PPP2R1A SUMO1 YWHAG
REACTOME	Signal Transduction	2598	2.13E-07	5.11E-05	SOX2 PPP2CA PTCH1 MAP2K1 CLTC CBL UBC HIST2H2BE SH3KBP1 MOV10 TRIB3 ITCH PML SFPQ ASH2L LATS1 PPP2R1A KAT2A CDH1 YWHAG
REACTOME	RNA Polymerase II Transcription	1196	2.01E-07	5.11E-05	RPA2 PPP2CA RPA1 UBC HIST2H2BE MOV10 PML NR0B2 ASH2L ITCH PPP2R1A KAT2A SUMO1 YWHAG
REACTOME	Generic Transcription Pathway	1074	5.32E-08	5.3E-05	RPA2 PPP2CA RPA1 UBC HIST2H2BE MOV10 PML NR0B2 ASH2L ITCH PPP2R1A KAT2A SUMO1 YWHAG
REACTOME	Negative regulation of FGFR3 signaling	23	1.92E-07	5.44E-05	PPP2R1A PPP2CA CBL UBC
REACTOME	Signaling by WNT	294	2.67E-07	5.77E-05	PPP2CA HIST2H2BE UBC CLTC MOV10 SOX2 ASH2L PPP2R1A
REACTOME	Negative regulation of FGFR1 signaling	26	3.22E-07	6.34E-05	PPP2R1A PPP2CA CBL UBC

REACTOME	Negative regulation of FGFR4 signaling	27	3.78E-07	6.81E-05	PPP2R1A PPP2CA CBL UBC
REACTOME	Negative regulation of FGFR2 signaling	28	4.4E-07	7.33E-05	PPP2R1A PPP2CA CBL UBC
REACTOME	Adaptive Immune System	743	5.53E-07	8.54E-05	PPP2CA CBL CLTC PDIA3 TRIB3 SH3KBP1 RAB7A UBC ITCH PPP2R1A CDH1
REACTOME	Clathrin-mediated endocytosis	138	6.32E-07	9.11E-05	CLTC CBL TFRC RAB5C SH3KBP1 UBC
REACTOME	G2/M DNA damage checkpoint	76	7.52E-07	0.000102	SUMO1 RPA2 YWHAG RPA1 HIST2H2BE
REACTOME	Processing of DNA double-strand break ends	79	9.13E-07	0.000104	SUMO1 RPA2 UBC RPA1 HIST2H2BE
REACTOME	DNA Double-Strand Break Repair	146	8.79E-07	0.000104	RPA2 RPA1 HIST2H2BE XRCC5 UBC SUMO1
REACTOME	Signaling by FGFR3	33	8.72E-07	0.000106	PPP2R1A PPP2CA CBL UBC
REACTOME	G2/M Checkpoints	149	9.91E-07	0.000107	RPA2 RPA1 HIST2H2BE UBC SUMO1 YWHAG
REACTOME	Transcriptional Regulation by TP53	359	1.2E-06	0.000123	RPA2 PPP2CA RPA1 MOV10 PML UBC PPP2R1A YWHAG
REACTOME	Signaling by FGFR4	36	1.25E-06	0.000123	PPP2R1A PPP2CA CBL UBC
REACTOME	Infectious disease	368	1.45E-06	0.000135	MAP2K1 CBL CLTC HMGA1 SH3KBP1 XRCC5 UBC CDH1
REACTOME	Regulation of TP53 Activity	160	1.5E-06	0.000135	RPA2 PPP2CA RPA1 PML UBC PPP2R1A
REACTOME	Negative regulation of MAPK pathway	40	1.93E-06	0.000167	PPP2R1A PPP2CA UBC MAP2K1
REACTOME	Signaling by FGFR1	42	2.35E-06	0.000196	PPP2R1A PPP2CA CBL UBC
REACTOME	Cargo recognition for clathrin-mediated endocytosis	98	2.67E-06	0.000206	TFRC CBL CLTC SH3KBP1 UBC
REACTOME	Formation of Incision Complex in GG-NER	43	2.59E-06	0.000206	SUMO1 RPA2 RPA1 UBC
REACTOME	InlB-mediated entry of Listeria monocytogenes into host cell	14	3.81E-06	0.000284	CBL SH3KBP1 UBC
REACTOME	Signaling by Receptor Tyrosine Kinases	433	4.83E-06	0.000349	PPP2CA CBL CLTC TRIB3 SH3KBP1 UBC ITCH PPP2R1A
REACTOME	HDR through Homologous Recombination (HR) or Single Strand Annealing (SSA)	112	5.14E-06	0.000359	SUMO1 RPA2 UBC RPA1 HIST2H2BE
REACTOME	TCF dependent signaling in response to WNT	199	5.31E-06	0.000359	ASH2L PPP2CA HIST2H2BE SOX2 UBC PPP2R1A
REACTOME	Translesion synthesis by REV1	16	5.84E-06	0.000372	RPA2 RPA1 UBC

REACTOME	Transcriptional regulation by RUNX1	202	5.78E-06	0.000372	HIST2H2BE MOV10 PML UBC ITCH ASH2L
REACTOME	Homology Directed Repair	118	6.64E-06	0.000411	SUMO1 RPA2 UBC RPA1 HIST2H2BE
REACTOME	Translesion synthesis by POLI	17	7.08E-06	0.000414	RPA2 RPA1 UBC
REACTOME	Translesion synthesis by POLK	17	7.08E-06	0.000414	RPA2 RPA1 UBC
KEGG	ENDOCYTOSIS	181	3.07E-06	0.000571	CLTC CBL TFRC RAB5C SH3KBP1 ITCH
REACTOME	Translesion Synthesis by POLH	19	1.01E-05	0.000573	RPA2 RPA1 UBC
REACTOME	G1/S Transition	130	1.07E-05	0.000591	PPP2R1A PPP2CA UBC RPA1 RPA2
REACTOME	Disease	1017	1.15E-05	0.000621	PPP2CA MAP2K1 CBL CLTC HMGA1 SH3KBP1 XRCC5 UBC PPP2R1A KAT2A CDH1
REACTOME	Negative regulation of MET activity	20	1.18E-05	0.000623	CBL SH3KBP1 UBC
REACTOME	MAP kinase activation	63	1.21E-05	0.000623	PPP2R1A PPP2CA UBC MAP2K1
REACTOME	Signaling by FGFR2	66	1.46E-05	0.000732	PPP2R1A PPP2CA CBL UBC
REACTOME	PIP3 activates AKT signaling	240	1.54E-05	0.00076	PPP2CA MOV10 TRIB3 PML UBC PPP2R1A
REACTOME	Interleukin-17 signaling	71	1.95E-05	0.000916	PPP2R1A PPP2CA UBC MAP2K1
REACTOME	Mitotic G1-G1/S phases	147	1.93E-05	0.000916	PPP2R1A PPP2CA UBC RPA1 RPA2
REACTOME	EGFR downregulation	24	2.08E-05	0.00094	CBL SH3KBP1 UBC
REACTOME	RAF activation	24	2.08E-05	0.00094	PPP2R1A PPP2CA MAP2K1
REACTOME	Cellular responses to stress	386	2.28E-05	0.001006	RPA2 HIST1H1A RPA1 HIST2H2BE MOV10 HMGA1 UBC
REACTOME	Gap-filling DNA repair synthesis and ligation in GG-NER	25	2.36E-05	0.001023	RPA2 RPA1 UBC
REACTOME	Signaling by FGFR	77	2.68E-05	0.001139	PPP2R1A PPP2CA CBL UBC
REACTOME	Cellular Senescence	160	2.9E-05	0.001208	HIST1H1A HIST2H2BE MOV10 HMGA1 UBC
REACTOME	Intracellular signaling by second messengers	271	3.06E-05	0.00125	PPP2CA MOV10 TRIB3 PML UBC PPP2R1A
GO	focal adhesion	402	2.29E-07	0.001361	PDIA3 TRIP6 MAP2K1 CBL CLTC HMGA1 SH3KBP1 FLOT2 YWHAG
GO	endocytic vesicle	59	2.1E-07	0.001361	SH3KBP1 RAB5C GIPC1 FLOT2 CD2AP
REACTOME	Toll Like Receptor 5 (TLR5) Cascade	85	3.96E-05	0.001505	PPP2R1A PPP2CA UBC MAP2K1
REACTOME	Toll Like Receptor 10 (TLR10) Cascade	85	3.96E-05	0.001505	PPP2R1A PPP2CA UBC MAP2K1
REACTOME	Global Genome Nucleotide Excision Repair (GG-NER)	84	3.78E-05	0.001516	SUMO1 RPA2 RPA1 UBC

REACTOME	MyD88 cascade initiated on plasma membrane	85	3.96E-05	0.001532	PPP2R1A PPP2CA UBC MAP2K1
GO	extracellular exosome	2154	3.47E-07	0.001546	PPP2CA KRT8 TFRC HIST2H2BE GIPC1 UBC CLTC PDIA3 CD2AP RAB5C RAB7A FLOT2 TUFM IQCB1 PPP2R1A YWHAG CDH1 ITCH
REACTOME	DNA Repair	291	4.56E-05	0.001651	RPA2 RPA1 HIST2H2BE XRCC5 UBC SUMO1
REACTOME	Recognition of DNA damage by PCNA-containing replication complex	31	4.58E-05	0.001651	RPA2 RPA1 UBC
REACTOME	Cell Cycle	591	4.55E-05	0.001671	RPA2 PPP2CA RPA1 HIST2H2BE UBC PPP2R1A SUMO1 YWHAG
GO	membrane organization	132	4.86E-07	0.001735	CLTC CBL TFRC SH3KBP1 UBC YWHAG
REACTOME	Termination of translesion DNA synthesis	32	5.04E-05	0.001789	RPA2 RPA1 UBC
REACTOME	TRAF6 mediated induction of NFkB and MAP kinases upon TLR7/8 or 9 activation	92	5.41E-05	0.001887	PPP2R1A PPP2CA UBC MAP2K1
REACTOME	MyD88 dependent cascade initiated on endosome	94	5.88E-05	0.001989	PPP2R1A PPP2CA UBC MAP2K1
REACTOME	Toll Like Receptor 7/8 (TLR7/8) Cascade	94	5.88E-05	0.001989	PPP2R1A PPP2CA UBC MAP2K1
REACTOME	Toll Like Receptor TLR6:TLR2 Cascade	95	6.13E-05	0.00201	PPP2R1A PPP2CA UBC MAP2K1
REACTOME	MyD88:Mal cascade initiated on plasma membrane	95	6.13E-05	0.00201	PPP2R1A PPP2CA UBC MAP2K1
REACTOME	Membrane Trafficking	618	6.24E-05	0.002014	CLTC CBL TFRC RAB5C SH3KBP1 UBC RAB7A YWHAG
REACTOME	Regulation of TP53 Degradation	36	7.22E-05	0.002082	PPP2R1A PPP2CA UBC
REACTOME	MyD88-independent TLR4 cascade	99	7.2E-05	0.002082	PPP2R1A PPP2CA UBC MAP2K1
REACTOME	TRIF(TICAM1)-mediated TLR4 signaling	99	7.2E-05	0.002106	PPP2R1A PPP2CA UBC MAP2K1
REACTOME	Toll Like Receptor 3 (TLR3) Cascade	98	6.92E-05	0.00211	PPP2R1A PPP2CA UBC MAP2K1
REACTOME	Toll Like Receptor 2 (TLR2) Cascade	98	6.92E-05	0.00211	PPP2R1A PPP2CA UBC MAP2K1
REACTOME	Cellular responses to external stimuli	462	7.12E-05	0.002135	RPA2 HIST1H1A RPA1 HIST2H2BE MOV10 HMGA1 UBC

REACTOME	Toll Like Receptor 9 (TLR9) Cascade	98	6.92E-05	0.00214	PPP2R1A PPP2CA UBC MAP2K1
REACTOME	Toll Like Receptor TLR1:TLR2 Cascade	98	6.92E-05	0.002171	PPP2R1A PPP2CA UBC MAP2K1
REACTOME	Regulation of TP53 Expression and Degradation	37	7.84E-05	0.002232	PPP2R1A PPP2CA UBC
REACTOME	Fanconi Anemia Pathway	38	8.5E-05	0.002388	RPA2 RPA1 UBC
REACTOME	Translesion synthesis by Y family DNA polymerases bypasses lesions on DNA template	39	9.19E-05	0.00255	RPA2 RPA1 UBC
REACTOME	Vesicle-mediated transport	655	9.36E-05	0.002563	CLTC CBL TFRC RAB5C SH3KBP1 UBC RAB7A YWHAG
GO	flotillin complex	9	8.86E-07	0.002633	CBL CDH1 FLOT2
REACTOME	PP2A-mediated dephosphorylation of key metabolic factors	7	0.000103	0.002659	PPP2R1A PPP2CA
REACTOME	2-LTR circle formation	7	0.000103	0.002659	XRCC5 HMGA1
REACTOME	Cell Cycle, Mitotic	487	9.9E-05	0.002678	RPA2 PPP2CA RPA1 HIST2H2BE UBC PPP2R1A YWHAG
REACTOME	Downregulation of ERBB4 signaling	7	0.000103	0.002691	ITCH UBC
REACTOME	Dual Incision in GG-NER	41	0.000107	0.002721	RPA2 RPA1 UBC
REACTOME	Cytokine Signaling in Immune system	664	0.000103	0.002724	PPP2CA MAP2K1 CBL SOX2 PML UBC PPP2R1A SUMO1
REACTOME	Nucleotide Excision Repair	110	0.000108	0.00273	SUMO1 RPA2 RPA1 UBC
REACTOME	Deactivation of the beta-catenin transactivating complex	42	0.000115	0.002826	SOX2 ASH2L UBC
REACTOME	Signaling by EGFR	42	0.000115	0.002826	CBL SH3KBP1 UBC
REACTOME	Cyclin D associated events in G1	43	0.000123	0.002966	PPP2R1A PPP2CA UBC
REACTOME	G1 Phase	43	0.000123	0.002966	PPP2R1A PPP2CA UBC
REACTOME	Diseases of signal transduction	359	0.000145	0.003441	PPP2CA MAP2K1 CBL UBC PPP2R1A KAT2A
GO	RNA binding	1415	1.53E-06	0.003908	PDIA3 UBC TRIP6 CLTC TSR1 TFRC CAVIN1 XRCC5 SFPQ MOV10 TUFM SUMO1 NXF1 YWHAG
REACTOME	Signaling by NOTCH	123	0.000167	0.003928	ITCH KAT2A MOV10 UBC
REACTOME	Integration of provirus	9	0.000176	0.003977	XRCC5 HMGA1
REACTOME	InlA-mediated entry of Listeria monocytogenes into host cells	9	0.000176	0.003977	CDH1 UBC

REACTOME	PTK6 Regulates RTKs and Their Effectors AKT1 and DOK1	9	0.000176	0.004019	CBL UBC
REACTOME	Regulation of PTEN localization	9	0.000176	0.004062	PML UBC
REACTOME	DNA Damage Bypass	49	0.000182	0.004069	RPA2 RPA1 UBC
REACTOME	Nonhomologous End-Joining (NHEJ)	50	0.000194	0.004254	SUMO1 XRCC5 HIST2H2BE
REACTOME	Toll Like Receptor 4 (TLR4) Cascade	128	0.000195	0.004254	PPP2R1A PPP2CA UBC MAP2K1
REACTOME	Initiation of Nuclear Envelope Reformation	10	0.00022	0.004539	PPP2R1A PPP2CA
REACTOME	Nuclear Envelope Reassembly	10	0.00022	0.004539	PPP2R1A PPP2CA
REACTOME	Removal of the Flap Intermediate from the C-strand	10	0.00022	0.004582	RPA2 RPA1
REACTOME	MASTL Facilitates Mitotic Progression	10	0.00022	0.004627	PPP2R1A PPP2CA
REACTOME	Signaling by Non-Receptor Tyrosine Kinases	52	0.000218	0.004666	CBL SFPQ UBC
REACTOME	Signaling by PTK6	52	0.000218	0.004666	CBL SFPQ UBC
REACTOME	Meiotic recombination	53	0.00023	0.004705	RPA2 RPA1 HIST2H2BE
REACTOME	Processive synthesis on the C-strand of the telomere	11	0.000269	0.005436	RPA2 RPA1
REACTOME	Recruitment and ATM-mediated phosphorylation of repair and signaling proteins at DNA double strand breaks	57	0.000286	0.005732	SUMO1 HIST2H2BE UBC
REACTOME	DNA Double Strand Break Response	58	0.000301	0.00598	SUMO1 HIST2H2BE UBC
REACTOME	DNA Damage/Telomere Stress Induced Senescence	59	0.000317	0.006233	HIST1H1A HIST2H2BE HMGA1
REACTOME	HSF1 activation	12	0.000322	0.006279	RPA2 RPA1
REACTOME	Telomere Maintenance	61	0.00035	0.006726	RPA2 RPA1 HIST2H2BE
REACTOME	MAPK family signaling cascades	272	0.000351	0.006726	PPP2R1A PPP2CA UBC MOV10 MAP2K1
REACTOME	RHO GTPase Effectors	274	0.000363	0.006894	PPP2R1A PPP2CA HIST2H2BE CDH1 YWHAG
GO	regulation of transcription, DNA-templated	898	3.51E-06	0.006957	MOV10 PPP2CA TRIP6 CBL HMGA1 SOX2 PML SFPQ CAVIN1 ASH2L PPP2R1A

GO	protein C-terminus binding	184	3.38E-06	0.006957	PPP2CA MAP2K1 PHB2 CD2AP XRCC5 SUMO1
REACTOME	ERKs are inactivated	13	0.00038	0.00709	PPP2R1A PPP2CA
REACTOME	Inhibition of replication initiation of damaged DNA by RB1/E2F1	13	0.00038	0.00709	PPP2R1A PPP2CA
REACTOME	RUNX1 regulates genes involved in megakaryocyte differentiation and platelet function	63	0.000385	0.007114	ASH2L HIST2H2BE MOV10
REACTOME	Truncations of AMER1 destabilize the destruction complex	14	0.000443	0.007204	PPP2R1A PPP2CA
REACTOME	AMER1 mutants destabilize the destruction complex	14	0.000443	0.007204	PPP2R1A PPP2CA
REACTOME	Toll-Like Receptors Cascades	154	0.000394	0.007226	PPP2R1A PPP2CA UBC MAP2K1
REACTOME	Innate Immune System	1027	0.000399	0.007252	PPP2CA MAP2K1 VAPA RAB5C XRCC5 UBC ITCH PPP2R1A RAB7A
REACTOME	Early Phase of HIV Life Cycle	14	0.000443	0.007259	XRCC5 HMGA1
REACTOME	Gap-filling DNA repair synthesis and ligation in TC-NER	64	0.000403	0.007265	RPA2 RPA1 UBC
REACTOME	AXIN mutants destabilize the destruction complex, activating WNT signaling	14	0.000443	0.007314	PPP2R1A PPP2CA
REACTOME	Mismatch repair (MMR) directed by MSH2:MSH6 (MutSalpha)	14	0.000443	0.007371	RPA2 RPA1
REACTOME	truncated APC mutants destabilize the destruction complex	14	0.000443	0.007428	PPP2R1A PPP2CA
REACTOME	Dual incision in TC-NER	65	0.000422	0.00748	RPA2 RPA1 UBC
REACTOME	Costimulation by the CD28 family	65	0.000422	0.00748	TRIB3 PPP2R1A PPP2CA
REACTOME	Mismatch repair (MMR) directed by MSH2:MSH3 (MutSbeta)	14	0.000443	0.007486	RPA2 RPA1
REACTOME	Removal of the Flap Intermediate	14	0.000443	0.007545	RPA2 RPA1
REACTOME	Signaling by Interleukins	447	0.000469	0.007576	PPP2CA MAP2K1 CBL SOX2 UBC PPP2R1A

REACTOME	APC truncation mutants have impaired AXIN binding	14	0.000443	0.007605	PPP2R1A PPP2CA
REACTOME	AXIN missense mutants destabilize the destruction complex	14	0.000443	0.007666	PPP2R1A PPP2CA
REACTOME	HDR through Homologous Recombination (HRR)	66	0.000441	0.007727	RPA2 RPA1 UBC
REACTOME	Misspliced GSK3beta mutants stabilize beta-catenin	15	0.00051	0.007775	PPP2R1A PPP2CA
REACTOME	Mismatch Repair	15	0.00051	0.007775	RPA2 RPA1
REACTOME	T41 mutants of beta-catenin aren't phosphorylated	15	0.00051	0.00783	PPP2R1A PPP2CA
REACTOME	SUMOylation of DNA damage response and repair proteins	70	0.000524	0.00788	SUMO1 PML RPA1
REACTOME	Apoptosis	166	0.000523	0.00788	HIST1H1A YWHAG CDH1 UBC
REACTOME	S45 mutants of beta-catenin aren't phosphorylated	15	0.00051	0.007886	PPP2R1A PPP2CA
REACTOME	phosphorylation site mutants of CTNNB1 are not targeted to the proteasome by the destruction complex	15	0.00051	0.007943	PPP2R1A PPP2CA
REACTOME	S33 mutants of beta-catenin aren't phosphorylated	15	0.00051	0.008	PPP2R1A PPP2CA
REACTOME	S37 mutants of beta-catenin aren't phosphorylated	15	0.00051	0.008059	PPP2R1A PPP2CA
REACTOME	Processive synthesis on the lagging strand	15	0.00051	0.008118	RPA2 RPA1
REACTOME	Signaling by NOTCH1	71	0.000547	0.008158	ITCH KAT2A UBC
REACTOME	Programmed Cell Death	169	0.00056	0.008294	HIST1H1A YWHAG CDH1 UBC
REACTOME	Clathrin derived vesicle budding	72	0.00057	0.008327	TFRC CLTC RAB5C
REACTOME	trans-Golgi Network Vesicle Budding	72	0.00057	0.008327	TFRC CLTC RAB5C
REACTOME	Formation of Senescence-Associated Heterochromatin Foci (SAHF)	16	0.000582	0.0084	HIST1H1A HMGA1

REACTOME	MAP3K8 (TPL2)-dependent MAPK1/3 activation	16	0.000582	0.0084	UBC MAP2K1
REACTOME	Beta-catenin phosphorylation cascade	17	0.000659	0.00932	PPP2R1A PPP2CA
REACTOME	Regulation of RUNX1 Expression and Activity	17	0.000659	0.00932	PML MOV10
REACTOME	Signaling by NTRK1 (TRKA)	76	0.000667	0.009374	PPP2R1A PPP2CA CLTC
REACTOME	Platelet sensitization by LDL	17	0.000659	0.009381	PPP2R1A PPP2CA
REACTOME	Signaling by MET	77	0.000693	0.009676	CBL SH3KBP1 UBC
REACTOME	Transcription-Coupled Nucleotide Excision Repair (TC-NER)	78	0.00072	0.00992	RPA2 RPA1 UBC
REACTOME	RNA Polymerase I Transcription	78	0.00072	0.00992	KAT2A HIST2H2BE CAVIN1
REACTOME	Constitutive Signaling by Ligand-Responsive EGFR Cancer Variants	18	0.00074	0.010011	CBL UBC
REACTOME	Signaling by EGFR in Cancer	18	0.00074	0.010011	CBL UBC
REACTOME	Signaling by Ligand-Responsive EGFR Variants in Cancer	18	0.00074	0.010074	CBL UBC
REACTOME	Degradation of beta-catenin by the destruction complex	82	0.000833	0.01119	PPP2R1A PPP2CA UBC
KEGG	OOCYTE_MEIOSIS	113	0.00012	0.011196	PPP2R1A PPP2CA YWHAG MAP2K1
REACTOME	Meiosis	83	0.000862	0.01152	RPA2 RPA1 HIST2H2BE
REACTOME	G2/M Transition	191	0.000884	0.011741	PPP2R1A PPP2CA YWHAG UBC
REACTOME	Hedgehog 'on' state	85	0.000924	0.011764	ITCH PTCH1 UBC
REACTOME	DNA Replication Pre-Initiation	85	0.000924	0.011764	RPA2 RPA1 UBC
REACTOME	Regulation of PLK1 Activity at G2/M Transition	85	0.000924	0.011834	PPP2R1A YWHAG UBC
REACTOME	M/G1 Transition	85	0.000924	0.011904	RPA2 RPA1 UBC
REACTOME	Mitotic G2-G2/M phases	193	0.000919	0.011975	PPP2R1A PPP2CA YWHAG UBC
REACTOME	Lagging Strand Synthesis	20	0.000917	0.011984	RPA2 RPA1
REACTOME	CTLA4 inhibitory signaling	20	0.000917	0.012021	PPP2R1A PPP2CA
REACTOME	PCNA-Dependent Long Patch Base Excision Repair	21	0.001012	0.012728	RPA2 RPA1
REACTOME	Regulation of signaling by CBL	21	0.001012	0.012728	CBL UBC
REACTOME	Chromosome Maintenance	88	0.001022	0.012783	RPA2 RPA1 HIST2H2BE

REACTOME	Negative regulation of the PI3K/AKT network	89	0.001056	0.013057	TRIB3 PPP2R1A PPP2CA
REACTOME	M Phase	347	0.001056	0.013057	PPP2R1A PPP2CA YWHAG HIST2H2BE UBC
REACTOME	Oxidative Stress Induced Senescence	90	0.001091	0.01341	HIST2H2BE MOV10 UBC
REACTOME	ERK/MAPK targets	22	0.001111	0.013509	PPP2R1A PPP2CA
REACTOME	E2F mediated regulation of DNA replication	22	0.001111	0.013509	PPP2R1A PPP2CA
REACTOME	Signaling by NTRKs	92	0.001162	0.013972	PPP2R1A PPP2CA CLTC
REACTOME	Regulation of TP53 Activity through Phosphorylation	92	0.001162	0.013972	RPA2 RPA1 UBC
REACTOME	DARPP-32 events	24	0.001324	0.015655	PPP2R1A PPP2CA
REACTOME	Telomere C-strand (Lagging Strand) Synthesis	24	0.001324	0.015655	RPA2 RPA1
REACTOME	Anchoring of the basal body to the plasma membrane	96	0.001314	0.01571	IQCB1 PPP2R1A YWHAG
REACTOME	RUNX1 regulates transcription of genes involved in differentiation of HSCs	97	0.001354	0.015922	ITCH HIST2H2BE UBC
REACTOME	Resolution of AP sites via the multiple-nucleotide patch replacement pathway	25	0.001437	0.016629	RPA2 RPA1
REACTOME	Cyclin A/B1/B2 associated events during G2/M transition	25	0.001437	0.016629	PPP2R1A PPP2CA
REACTOME	Nuclear Events (kinase and transcription factor activation)	25	0.001437	0.016718	PPP2R1A PPP2CA
REACTOME	Mitotic Prophase	100	0.001478	0.017011	PPP2R1A PPP2CA HIST2H2BE
REACTOME	Calnexin/calreticulin cycle	26	0.001554	0.017798	PDIA3 UBC
GO	error-prone translesion synthesis	21	1.38E-05	0.018883	RPA2 RPA1 UBC
GO	negative regulation of DNA binding transcription factor activity	65	1.37E-05	0.018883	PHB2 SUMO1 NR0B2 PTCH1
GO	protein localization to chromosome	3	1.48E-05	0.018896	RPA2 RPA1
GO	cytosol	4995	1.14E-05	0.019158	KRT8 CLTC HMGA1 PML IQCB1 PPP2R1A GIPC1 HIST2H2BE LATS1 PPP2CA TRIP6 CBL TSR1 TRIB3 ITCH SH3KBP1 CAVIN1 NXF1 RAB7A

					MAP2K1 MOV10 SOX2 XRCC5 UBC YWHAG
GO	error-free translesion synthesis	20	1.18E-05	0.019158	RPA2 RPA1 UBC
REACTOME	RAF/MAP kinase cascade	228	0.001699	0.019353	PPP2R1A PPP2CA UBC MAP2K1
REACTOME	SUMO E3 ligases SUMOylate target proteins	106	0.001747	0.019789	SUMO1 PML RPA1
REACTOME	Developmental Biology	1023	0.001816	0.020472	KRT8 MAP2K1 CLTC HIST2H2BE SOX2 SH3KBP1 UBC ASH2L
REACTOME	Reproduction	108	0.001843	0.020554	RPA2 RPA1 HIST2H2BE
REACTOME	MAPK1/MAPK3 signaling	233	0.001839	0.020554	PPP2R1A PPP2CA UBC MAP2K1
REACTOME	Signaling by Rho GTPases	402	0.002019	0.022405	PPP2R1A PPP2CA HIST2H2BE CDH1 YWHAG
REACTOME	SUMOylation	112	0.002044	0.022456	SUMO1 PML RPA1
REACTOME	Hedgehog 'off' state	112	0.002044	0.022456	ITCH PTCH1 UBC
REACTOME	Extension of Telomeres	30	0.002069	0.02261	RPA2 RPA1
REACTOME	Signaling by the B Cell Receptor (BCR)	113	0.002097	0.022801	CBL SH3KBP1 UBC
REACTOME	Disassembly of the destruction complex and recruitment of AXIN to the membrane	31	0.002208	0.023425	PPP2R1A PPP2CA
REACTOME	Pre-NOTCH Transcription and Translation	31	0.002208	0.023425	KAT2A MOV10
REACTOME	MAPK targets/ Nuclear events mediated by MAP kinases	31	0.002208	0.02354	PPP2R1A PPP2CA
REACTOME	TGF-beta receptor signaling activates SMADs	31	0.002208	0.023657	CBL UBC
REACTOME	Activated NOTCH1 Transmits Signal to the Nucleus	31	0.002208	0.023774	ITCH UBC
GO	nucleoplasm	3162	2.14E-05	0.023872	RPA2 KRT8 RPA1 TSR1 HIST2H2BE HMGA1 SOX2 ITCH PML XRCC5 SFPQ CAVIN1 NR0B2 UBC IQCB1 ASH2L KAT2A SUMO1 NXF1
GO	nucleotide-excision repair, DNA gap filling	24	2.08E-05	0.023872	RPA2 RPA1 UBC
REACTOME	DNA strand elongation	32	0.002352	0.024829	RPA2 RPA1
BIOCARTA	CHREBP2_PATHWAY	42	0.000115	0.024937	PPP2R1A PPP2CA YWHAG
REACTOME	Synthesis of DNA	119	0.002429	0.025518	RPA2 RPA1 UBC
REACTOME	Activation of the pre-replicative complex	33	0.0025	0.025765	RPA2 RPA1
REACTOME	Oncogene Induced Senescence	33	0.0025	0.025765	MOV10 UBC

REACTOME	Antigen activates B Cell Receptor (BCR) leading to generation of second messengers	33	0.0025	0.025888	CBL SH3KBP1
REACTOME	Post-translational protein modification	1331	0.002495	0.026013	PDIA3 RPA1 HIST2H2BE RAB5C PML UBC KAT2A SUMO1 RAB7A
REACTOME	Negative regulators of DDX58/IFIH1 signaling	34	0.002653	0.027078	ITCH UBC
REACTOME	Signaling by WNT in cancer	34	0.002653	0.027078	PPP2R1A PPP2CA
REACTOME	NOD1/2 Signaling Pathway	35	0.00281	0.02841	ITCH UBC
REACTOME	N-glycan trimming in the ER and Calnexin/Calreticulin cycle	35	0.00281	0.02841	PDIA3 UBC
GO	second-messenger-mediated signaling	4	2.96E-05	0.029351	PPP2R1A PPP2CA
GO	PML body organization	4	2.96E-05	0.029351	PML SUMO1
REACTOME	DNA Replication	127	0.002921	0.029398	RPA2 RPA1 UBC
BIOCARTA	SPRY_PATHWAY	18	0.00074	0.029878	CBL MAP2K1
BIOCARTA	TGFB_PATHWAY	19	0.000826	0.029878	CDH1 MAP2K1
REACTOME	HDR through Single Strand Annealing (SSA)	37	0.003136	0.030986	RPA2 RPA1
REACTOME	Base Excision Repair	37	0.003136	0.030986	RPA2 RPA1
REACTOME	Resolution of Abasic Sites (AP sites)	37	0.003136	0.031128	RPA2 RPA1
REACTOME	Activation of ATR in response to replication stress	37	0.003136	0.031271	RPA2 RPA1
BIOCARTA	TEL_PATHWAY	18	0.00074	0.032126	PPP2CA XRCC5
KEGG	LONG_TERM_DEPRESSION	70	0.000524	0.032511	PPP2R1A PPP2CA MAP2K1
REACTOME	Presynaptic phase of homologous DNA pairing and strand exchange	39	0.003479	0.034221	RPA2 RPA1
GO	nucleotide-excision repair, preincision complex assembly	29	3.73E-05	0.035052	RPA2 RPA1 UBC
REACTOME	PTEN Regulation	138	0.003691	0.036137	PML MOV10 UBC
KEGG	PATHWAYS_IN_CANCER	325	0.000788	0.036635	CBL PML CDH1 PTCH1 MAP2K1
REACTOME	Signaling by ERBB4	41	0.003839	0.037421	ITCH UBC
REACTOME	Homologous DNA Pairing and Strand Exchange	42	0.004025	0.03906	RPA2 RPA1
REACTOME	Beta-catenin independent WNT signaling	143	0.004078	0.039392	CLTC MOV10 UBC
BIOCARTA	PML_PATHWAY	17	0.000659	0.040157	PML SUMO1

BIOCARTA	CBL_PATHWAY	13	0.00038	0.041239	CBL SH3KBP1
REACTOME	TBC/RABGAPs	44	0.00441	0.042226	RAB5C RAB7A
REACTOME	HIV Life Cycle	147	0.004404	0.042226	XRCC5 HMGA1 UBC
REACTOME	Entry of Influenza Virion into Host Cell via Endocytosis	2	0.004495	0.042664	CLTC
REACTOME	Signaling by Hedgehog	148	0.004488	0.042664	ITCH PTCH1 UBC
REACTOME	NOTCH1 Intracellular Domain Regulates Transcription	45	0.004608	0.043549	KAT2A UBC
KEGG	MISMATCH_REPAIR	23	0.001215	0.045209	RPA2 RPA1
REACTOME	Interleukin-3, 5 and GM-CSF signaling	47	0.005018	0.047006	CBL UBC
REACTOME	Pre-NOTCH Expression and Processing	47	0.005018	0.047006	KAT2A MOV10
REACTOME	Signaling by NOTCH3	48	0.005228	0.048769	KAT2A UBC

Table S3: Gene set enrichment analyses of genes outside of the PH and Fibrosis Networks interacting with miR-130/301 target genes and factors associated with heritable PAH. Pathways are listed in order of lowest to highest FDR p-value (Q).

Source	Annotation	Size	P	Q	Genes
GO	protein binding	9409	3.78E-11	6.75E-07	KRT8 RPA1 CLTC VAPA HMGA1 PML SFPQ TUFM IQCB1 PPP2R1A RPA2 PTCH1 GIPC1 CD2AP FLOT2 LATS1 ASH2L CDH1 PPP2CA HIST1H1A TRIP6 CBL TFRC TRIB3 RAB5C SH3KBP1 CAVIN1 NR0B2 KAT2A SUMO1 NXF1 RAB7A ITCH PDIA3 LMO4 MAP2K1 PHB2 MOV10 SOX2 XRCC5 UBC YWHAG
REACTOME	Gene expression (Transcription)	1330	1.05E-07	4.54E-05	RPA2 PPP2CA RPA1 UBC HIST2H2BE CAVIN1 PML MOV10 NR0B2 ASH2L ITCH PPP2R1A KAT2A SUMO1 YWHAG
REACTOME	Listeria monocytogenes entry into host cells	19	8.45E-08	4.54E-05	CBL SH3KBP1 CDH1 UBC
REACTOME	Immune System	1945	7.35E-08	4.57E-05	PDIA3 PPP2CA MAP2K1 SH3KBP1 CBL CLTC VAPA TRIB3 RAB5C PML XRCC5 RAB7A UBC ITCH PPP2R1A SOX2 SUMO1 CDH1
REACTOME	Spry regulation of FGF signaling	14	2.2E-08	4.76E-05	PPP2R1A PPP2CA CBL UBC
REACTOME	Cell Cycle Checkpoints	270	1.39E-07	5.02E-05	RPA2 PPP2CA RPA1 HIST2H2BE UBC PPP2R1A SUMO1 YWHAG
REACTOME	Signal Transduction	2598	2.13E-07	5.11E-05	SOX2 PPP2CA PTCH1 MAP2K1 CLTC CBL UBC HIST2H2BE SH3KBP1 MOV10 TRIB3 ITCH PML SFPQ ASH2L LATS1 PPP2R1A KAT2A CDH1 YWHAG
REACTOME	RNA Polymerase II Transcription	1196	2.01E-07	5.11E-05	RPA2 PPP2CA RPA1 UBC HIST2H2BE MOV10 PML NR0B2 ASH2L ITCH PPP2R1A KAT2A SUMO1 YWHAG
REACTOME	Generic Transcription Pathway	1074	5.32E-08	5.3E-05	RPA2 PPP2CA RPA1 UBC HIST2H2BE MOV10 PML NR0B2 ASH2L ITCH PPP2R1A KAT2A SUMO1 YWHAG
REACTOME	Negative regulation of FGFR3 signaling	23	1.92E-07	5.44E-05	PPP2R1A PPP2CA CBL UBC
REACTOME	Signaling by WNT	294	2.67E-07	5.77E-05	PPP2CA HIST2H2BE UBC CLTC MOV10 SOX2 ASH2L PPP2R1A
REACTOME	Negative regulation of FGFR1 signaling	26	3.22E-07	6.34E-05	PPP2R1A PPP2CA CBL UBC

REACTOME	Negative regulation of FGFR4 signaling	27	3.78E-07	6.81E-05	PPP2R1A PPP2CA CBL UBC
REACTOME	Negative regulation of FGFR2 signaling	28	4.4E-07	7.33E-05	PPP2R1A PPP2CA CBL UBC
REACTOME	Adaptive Immune System	743	5.53E-07	8.54E-05	PPP2CA CBL CLTC PDIA3 TRIB3 SH3KBP1 RAB7A UBC ITCH PPP2R1A CDH1
REACTOME	Clathrin-mediated endocytosis	138	6.32E-07	9.11E-05	CLTC CBL TFRC RAB5C SH3KBP1 UBC
REACTOME	G2/M DNA damage checkpoint	76	7.52E-07	0.000102	SUMO1 RPA2 YWHAG RPA1 HIST2H2BE
REACTOME	Processing of DNA double-strand break ends	79	9.13E-07	0.000104	SUMO1 RPA2 UBC RPA1 HIST2H2BE
REACTOME	DNA Double-Strand Break Repair	146	8.79E-07	0.000104	RPA2 RPA1 HIST2H2BE XRCC5 UBC SUMO1
REACTOME	Signaling by FGFR3	33	8.72E-07	0.000106	PPP2R1A PPP2CA CBL UBC
REACTOME	G2/M Checkpoints	149	9.91E-07	0.000107	RPA2 RPA1 HIST2H2BE UBC SUMO1 YWHAG
REACTOME	Transcriptional Regulation by TP53	359	1.2E-06	0.000123	RPA2 PPP2CA RPA1 MOV10 PML UBC PPP2R1A YWHAG
REACTOME	Signaling by FGFR4	36	1.25E-06	0.000123	PPP2R1A PPP2CA CBL UBC
REACTOME	Infectious disease	368	1.45E-06	0.000135	MAP2K1 CBL CLTC HMGA1 SH3KBP1 XRCC5 UBC CDH1
REACTOME	Regulation of TP53 Activity	160	1.5E-06	0.000135	RPA2 PPP2CA RPA1 PML UBC PPP2R1A
REACTOME	Negative regulation of MAPK pathway	40	1.93E-06	0.000167	PPP2R1A PPP2CA UBC MAP2K1
REACTOME	Signaling by FGFR1	42	2.35E-06	0.000196	PPP2R1A PPP2CA CBL UBC
REACTOME	Cargo recognition for clathrin-mediated endocytosis	98	2.67E-06	0.000206	TFRC CBL CLTC SH3KBP1 UBC
REACTOME	Formation of Incision Complex in GG-NER	43	2.59E-06	0.000206	SUMO1 RPA2 RPA1 UBC
REACTOME	InlB-mediated entry of Listeria monocytogenes into host cell	14	3.81E-06	0.000284	CBL SH3KBP1 UBC
REACTOME	Signaling by Receptor Tyrosine Kinases	433	4.83E-06	0.000349	PPP2CA CBL CLTC TRIB3 SH3KBP1 UBC ITCH PPP2R1A
REACTOME	HDR through Homologous Recombination (HR) or Single Strand Annealing (SSA)	112	5.14E-06	0.000359	SUMO1 RPA2 UBC RPA1 HIST2H2BE
REACTOME	TCF dependent signaling in response to WNT	199	5.31E-06	0.000359	ASH2L PPP2CA HIST2H2BE SOX2 UBC PPP2R1A
REACTOME	Translesion synthesis by REV1	16	5.84E-06	0.000372	RPA2 RPA1 UBC

REACTOME	Transcriptional regulation by RUNX1	202	5.78E-06	0.000372	HIST2H2BE MOV10 PML UBC ITCH ASH2L
REACTOME	Homology Directed Repair	118	6.64E-06	0.000411	SUMO1 RPA2 UBC RPA1 HIST2H2BE
REACTOME	Translesion synthesis by POLI	17	7.08E-06	0.000414	RPA2 RPA1 UBC
REACTOME	Translesion synthesis by POLK	17	7.08E-06	0.000414	RPA2 RPA1 UBC
KEGG	ENDOCYTOSIS	181	3.07E-06	0.000571	CLTC CBL TFRC RAB5C SH3KBP1 ITCH
REACTOME	Translesion Synthesis by POLH	19	1.01E-05	0.000573	RPA2 RPA1 UBC
REACTOME	G1/S Transition	130	1.07E-05	0.000591	PPP2R1A PPP2CA UBC RPA1 RPA2
REACTOME	Disease	1017	1.15E-05	0.000621	PPP2CA MAP2K1 CBL CLTC HMGA1 SH3KBP1 XRCC5 UBC PPP2R1A KAT2A CDH1
REACTOME	Negative regulation of MET activity	20	1.18E-05	0.000623	CBL SH3KBP1 UBC
REACTOME	MAP kinase activation	63	1.21E-05	0.000623	PPP2R1A PPP2CA UBC MAP2K1
REACTOME	Signaling by FGFR2	66	1.46E-05	0.000732	PPP2R1A PPP2CA CBL UBC
REACTOME	PIP3 activates AKT signaling	240	1.54E-05	0.00076	PPP2CA MOV10 TRIB3 PML UBC PPP2R1A
REACTOME	Interleukin-17 signaling	71	1.95E-05	0.000916	PPP2R1A PPP2CA UBC MAP2K1
REACTOME	Mitotic G1-G1/S phases	147	1.93E-05	0.000916	PPP2R1A PPP2CA UBC RPA1 RPA2
REACTOME	EGFR downregulation	24	2.08E-05	0.00094	CBL SH3KBP1 UBC
REACTOME	RAF activation	24	2.08E-05	0.00094	PPP2R1A PPP2CA MAP2K1
REACTOME	Cellular responses to stress	386	2.28E-05	0.001006	RPA2 HIST1H1A RPA1 HIST2H2BE MOV10 HMGA1 UBC
REACTOME	Gap-filling DNA repair synthesis and ligation in GG-NER	25	2.36E-05	0.001023	RPA2 RPA1 UBC
REACTOME	Signaling by FGFR	77	2.68E-05	0.001139	PPP2R1A PPP2CA CBL UBC
REACTOME	Cellular Senescence	160	2.9E-05	0.001208	HIST1H1A HIST2H2BE MOV10 HMGA1 UBC
REACTOME	Intracellular signaling by second messengers	271	3.06E-05	0.00125	PPP2CA MOV10 TRIB3 PML UBC PPP2R1A
GO	focal adhesion	402	2.29E-07	0.001361	PDIA3 TRIP6 MAP2K1 CBL CLTC HMGA1 SH3KBP1 FLOT2 YWHAG
GO	endocytic vesicle	59	2.1E-07	0.001361	SH3KBP1 RAB5C GIPC1 FLOT2 CD2AP
REACTOME	Toll Like Receptor 5 (TLR5) Cascade	85	3.96E-05	0.001505	PPP2R1A PPP2CA UBC MAP2K1
REACTOME	Toll Like Receptor 10 (TLR10) Cascade	85	3.96E-05	0.001505	PPP2R1A PPP2CA UBC MAP2K1

REACTOME	Global Genome Nucleotide Excision Repair (GG-NER)	84	3.78E-05	0.001516	SUMO1 RPA2 RPA1 UBC
REACTOME	MyD88 cascade initiated on plasma membrane	85	3.96E-05	0.001532	PPP2R1A PPP2CA UBC MAP2K1
GO	extracellular exosome	2154	3.47E-07	0.001546	PPP2CA KRT8 TFRC HIST2H2BE GIPC1 UBC CLTC PDIA3 CD2AP RAB5C RAB7A FLOT2 TUFM IQCB1 PPP2R1A YWHAG CDH1 ITCH
REACTOME	DNA Repair	291	4.56E-05	0.001651	RPA2 RPA1 HIST2H2BE XRCC5 UBC SUMO1
REACTOME	Recognition of DNA damage by PCNA-containing replication complex	31	4.58E-05	0.001651	RPA2 RPA1 UBC
REACTOME	Cell Cycle	591	4.55E-05	0.001671	RPA2 PPP2CA RPA1 HIST2H2BE UBC PPP2R1A SUMO1 YWHAG
GO	membrane organization	132	4.86E-07	0.001735	CLTC CBL TFRC SH3KBP1 UBC YWHAG
REACTOME	Termination of translesion DNA synthesis	32	5.04E-05	0.001789	RPA2 RPA1 UBC
REACTOME	TRAF6 mediated induction of NFkB and MAP kinases upon TLR7/8 or 9 activation	92	5.41E-05	0.001887	PPP2R1A PPP2CA UBC MAP2K1
REACTOME	MyD88 dependent cascade initiated on endosome	94	5.88E-05	0.001989	PPP2R1A PPP2CA UBC MAP2K1
REACTOME	Toll Like Receptor 7/8 (TLR7/8) Cascade	94	5.88E-05	0.001989	PPP2R1A PPP2CA UBC MAP2K1
REACTOME	Toll Like Receptor TLR6:TLR2 Cascade	95	6.13E-05	0.00201	PPP2R1A PPP2CA UBC MAP2K1
REACTOME	MyD88:Mal cascade initiated on plasma membrane	95	6.13E-05	0.00201	PPP2R1A PPP2CA UBC MAP2K1
REACTOME	Membrane Trafficking	618	6.24E-05	0.002014	CLTC CBL TFRC RAB5C SH3KBP1 UBC RAB7A YWHAG
REACTOME	Regulation of TP53 Degradation	36	7.22E-05	0.002082	PPP2R1A PPP2CA UBC
REACTOME	MyD88-independent TLR4 cascade	99	7.2E-05	0.002082	PPP2R1A PPP2CA UBC MAP2K1
REACTOME	TRIF(TICAM1)-mediated TLR4 signaling	99	7.2E-05	0.002106	PPP2R1A PPP2CA UBC MAP2K1
REACTOME	Toll Like Receptor 3 (TLR3) Cascade	98	6.92E-05	0.00211	PPP2R1A PPP2CA UBC MAP2K1

REACTOME	Toll Like Receptor 2 (TLR2) Cascade	98	6.92E-05	0.00211	PPP2R1A PPP2CA UBC MAP2K1
REACTOME	Cellular responses to external stimuli	462	7.12E-05	0.002135	RPA2 HIST1H1A RPA1 HIST2H2BE MOV10 HMGA1 UBC
REACTOME	Toll Like Receptor 9 (TLR9) Cascade	98	6.92E-05	0.00214	PPP2R1A PPP2CA UBC MAP2K1
REACTOME	Toll Like Receptor TLR1:TLR2 Cascade	98	6.92E-05	0.002171	PPP2R1A PPP2CA UBC MAP2K1
REACTOME	Regulation of TP53 Expression and Degradation	37	7.84E-05	0.002232	PPP2R1A PPP2CA UBC
REACTOME	Fanconi Anemia Pathway	38	8.5E-05	0.002388	RPA2 RPA1 UBC
REACTOME	Translesion synthesis by Y family DNA polymerases bypasses lesions on DNA template	39	9.19E-05	0.00255	RPA2 RPA1 UBC
REACTOME	Vesicle-mediated transport	655	9.36E-05	0.002563	CLTC CBL TFRC RAB5C SH3KBP1 UBC RAB7A YWHAG
GO	flotillin complex	9	8.86E-07	0.002633	CBL CDH1 FLOT2
REACTOME	PP2A-mediated dephosphorylation of key metabolic factors	7	0.000103	0.002659	PPP2R1A PPP2CA
REACTOME	2-LTR circle formation	7	0.000103	0.002659	XRCC5 HMGA1
REACTOME	Cell Cycle, Mitotic	487	9.9E-05	0.002678	RPA2 PPP2CA RPA1 HIST2H2BE UBC PPP2R1A YWHAG
REACTOME	Downregulation of ERBB4 signaling	7	0.000103	0.002691	ITCH UBC
REACTOME	Dual Incision in GG-NER	41	0.000107	0.002721	RPA2 RPA1 UBC
REACTOME	Cytokine Signaling in Immune system	664	0.000103	0.002724	PPP2CA MAP2K1 CBL SOX2 PML UBC PPP2R1A SUMO1
REACTOME	Nucleotide Excision Repair	110	0.000108	0.00273	SUMO1 RPA2 RPA1 UBC
REACTOME	Deactivation of the beta-catenin transactivating complex	42	0.000115	0.002826	SOX2 ASH2L UBC
REACTOME	Signaling by EGFR	42	0.000115	0.002826	CBL SH3KBP1 UBC
REACTOME	Cyclin D associated events in G1	43	0.000123	0.002966	PPP2R1A PPP2CA UBC
REACTOME	G1 Phase	43	0.000123	0.002966	PPP2R1A PPP2CA UBC
REACTOME	Diseases of signal transduction	359	0.000145	0.003441	PPP2CA MAP2K1 CBL UBC PPP2R1A KAT2A
GO	RNA binding	1415	1.53E-06	0.003908	PDIA3 UBC TRIP6 CLTC TSR1 TFRC CAVIN1 XRCC5 SFPQ MOV10 TUFM SUMO1 NXF1 YWHAG
REACTOME	Signaling by NOTCH	123	0.000167	0.003928	ITCH KAT2A MOV10 UBC
REACTOME	Integration of provirus	9	0.000176	0.003977	XRCC5 HMGA1

REACTOME	InlA-mediated entry of Listeria monocytogenes into host cells	9	0.000176	0.003977	CDH1 UBC
REACTOME	PTK6 Regulates RTKs and Their Effectors AKT1 and DOK1	9	0.000176	0.004019	CBL UBC
REACTOME	Regulation of PTEN localization	9	0.000176	0.004062	PML UBC
REACTOME	DNA Damage Bypass	49	0.000182	0.004069	RPA2 RPA1 UBC
REACTOME	Nonhomologous End-Joining (NHEJ)	50	0.000194	0.004254	SUMO1 XRCC5 HIST2H2BE
REACTOME	Toll Like Receptor 4 (TLR4) Cascade	128	0.000195	0.004254	PPP2R1A PPP2CA UBC MAP2K1
REACTOME	Initiation of Nuclear Envelope Reformation	10	0.00022	0.004539	PPP2R1A PPP2CA
REACTOME	Nuclear Envelope Reassembly	10	0.00022	0.004539	PPP2R1A PPP2CA
REACTOME	Removal of the Flap Intermediate from the C-strand	10	0.00022	0.004582	RPA2 RPA1
REACTOME	MASTL Facilitates Mitotic Progression	10	0.00022	0.004627	PPP2R1A PPP2CA
REACTOME	Signaling by Non-Receptor Tyrosine Kinases	52	0.000218	0.004666	CBL SFPQ UBC
REACTOME	Signaling by PTK6	52	0.000218	0.004666	CBL SFPQ UBC
REACTOME	Meiotic recombination	53	0.00023	0.004705	RPA2 RPA1 HIST2H2BE
REACTOME	Processive synthesis on the C-strand of the telomere	11	0.000269	0.005436	RPA2 RPA1
REACTOME	Recruitment and ATM-mediated phosphorylation of repair and signaling proteins at DNA double strand breaks	57	0.000286	0.005732	SUMO1 HIST2H2BE UBC
REACTOME	DNA Double Strand Break Response	58	0.000301	0.00598	SUMO1 HIST2H2BE UBC
REACTOME	DNA Damage/Telomere Stress Induced Senescence	59	0.000317	0.006233	HIST1H1A HIST2H2BE HMGA1
REACTOME	HSF1 activation	12	0.000322	0.006279	RPA2 RPA1
REACTOME	Telomere Maintenance	61	0.00035	0.006726	RPA2 RPA1 HIST2H2BE
REACTOME	MAPK family signaling cascades	272	0.000351	0.006726	PPP2R1A PPP2CA UBC MOV10 MAP2K1
REACTOME	RHO GTPase Effectors	274	0.000363	0.006894	PPP2R1A PPP2CA HIST2H2BE CDH1 YWHAG

GO	regulation of transcription, DNA-templated	898	3.51E-06	0.006957	MOV10 PPP2CA TRIP6 CBL HMGA1 SOX2 PML SFPQ CAVIN1 ASH2L PPP2R1A
GO	protein C-terminus binding	184	3.38E-06	0.006957	PPP2CA MAP2K1 PHB2 CD2AP XRCC5 SUMO1
REACTOME	ERKs are inactivated	13	0.00038	0.00709	PPP2R1A PPP2CA
REACTOME	Inhibition of replication initiation of damaged DNA by RB1/E2F1	13	0.00038	0.00709	PPP2R1A PPP2CA
REACTOME	RUNX1 regulates genes involved in megakaryocyte differentiation and platelet function	63	0.000385	0.007114	ASH2L HIST2H2BE MOV10
REACTOME	Truncations of AMER1 destabilize the destruction complex	14	0.000443	0.007204	PPP2R1A PPP2CA
REACTOME	AMER1 mutants destabilize the destruction complex	14	0.000443	0.007204	PPP2R1A PPP2CA
REACTOME	Toll-Like Receptors Cascades	154	0.000394	0.007226	PPP2R1A PPP2CA UBC MAP2K1
REACTOME	Innate Immune System	1027	0.000399	0.007252	PPP2CA MAP2K1 VAPA RAB5C XRCC5 UBC ITCH PPP2R1A RAB7A
REACTOME	Early Phase of HIV Life Cycle	14	0.000443	0.007259	XRCC5 HMGA1
REACTOME	Gap-filling DNA repair synthesis and ligation in TC-NER	64	0.000403	0.007265	RPA2 RPA1 UBC
REACTOME	AXIN mutants destabilize the destruction complex, activating WNT signaling	14	0.000443	0.007314	PPP2R1A PPP2CA
REACTOME	Mismatch repair (MMR) directed by MSH2:MSH6 (MutSalpha)	14	0.000443	0.007371	RPA2 RPA1
REACTOME	truncated APC mutants destabilize the destruction complex	14	0.000443	0.007428	PPP2R1A PPP2CA
REACTOME	Dual incision in TC-NER	65	0.000422	0.00748	RPA2 RPA1 UBC
REACTOME	Costimulation by the CD28 family	65	0.000422	0.00748	TRIB3 PPP2R1A PPP2CA
REACTOME	Mismatch repair (MMR) directed by MSH2:MSH3 (MutSbeta)	14	0.000443	0.007486	RPA2 RPA1
REACTOME	Removal of the Flap Intermediate	14	0.000443	0.007545	RPA2 RPA1

REACTOME	Signaling by Interleukins	447	0.000469	0.007576	PPP2CA MAP2K1 CBL SOX2 UBC PPP2R1A
REACTOME	APC truncation mutants have impaired AXIN binding	14	0.000443	0.007605	PPP2R1A PPP2CA
REACTOME	AXIN missense mutants destabilize the destruction complex	14	0.000443	0.007666	PPP2R1A PPP2CA
REACTOME	HDR through Homologous Recombination (HRR)	66	0.000441	0.007727	RPA2 RPA1 UBC
REACTOME	Misspliced GSK3beta mutants stabilize beta-catenin	15	0.00051	0.007775	PPP2R1A PPP2CA
REACTOME	Mismatch Repair	15	0.00051	0.007775	RPA2 RPA1
REACTOME	T41 mutants of beta-catenin aren't phosphorylated	15	0.00051	0.00783	PPP2R1A PPP2CA
REACTOME	SUMOylation of DNA damage response and repair proteins	70	0.000524	0.00788	SUMO1 PML RPA1
REACTOME	Apoptosis	166	0.000523	0.00788	HIST1H1A YWHAG CDH1 UBC
REACTOME	S45 mutants of beta-catenin aren't phosphorylated	15	0.00051	0.007886	PPP2R1A PPP2CA
REACTOME	phosphorylation site mutants of CTNNB1 are not targeted to the proteasome by the destruction complex	15	0.00051	0.007943	PPP2R1A PPP2CA
REACTOME	S33 mutants of beta-catenin aren't phosphorylated	15	0.00051	0.008	PPP2R1A PPP2CA
REACTOME	S37 mutants of beta-catenin aren't phosphorylated	15	0.00051	0.008059	PPP2R1A PPP2CA
REACTOME	Processive synthesis on the lagging strand	15	0.00051	0.008118	RPA2 RPA1
REACTOME	Signaling by NOTCH1	71	0.000547	0.008158	ITCH KAT2A UBC
REACTOME	Programmed Cell Death	169	0.00056	0.008294	HIST1H1A YWHAG CDH1 UBC
REACTOME	Clathrin derived vesicle budding	72	0.00057	0.008327	TFRC CLTC RAB5C
REACTOME	trans-Golgi Network Vesicle Budding	72	0.00057	0.008327	TFRC CLTC RAB5C
REACTOME	Formation of Senescence-Associated Heterochromatin Foci (SAHF)	16	0.000582	0.0084	HIST1H1A HMGA1

REACTOME	MAP3K8 (TPL2)-dependent MAPK1/3 activation	16	0.000582	0.0084	UBC MAP2K1
REACTOME	Beta-catenin phosphorylation cascade	17	0.000659	0.00932	PPP2R1A PPP2CA
REACTOME	Regulation of RUNX1 Expression and Activity	17	0.000659	0.00932	PML MOV10
REACTOME	Signaling by NTRK1 (TRKA)	76	0.000667	0.009374	PPP2R1A PPP2CA CLTC
REACTOME	Platelet sensitization by LDL	17	0.000659	0.009381	PPP2R1A PPP2CA
REACTOME	Signaling by MET	77	0.000693	0.009676	CBL SH3KBP1 UBC
REACTOME	Transcription-Coupled Nucleotide Excision Repair (TC-NER)	78	0.00072	0.00992	RPA2 RPA1 UBC
REACTOME	RNA Polymerase I Transcription	78	0.00072	0.00992	KAT2A HIST2H2BE CAVIN1
REACTOME	Constitutive Signaling by Ligand-Responsive EGFR Cancer Variants	18	0.00074	0.010011	CBL UBC
REACTOME	Signaling by EGFR in Cancer	18	0.00074	0.010011	CBL UBC
REACTOME	Signaling by Ligand-Responsive EGFR Variants in Cancer	18	0.00074	0.010074	CBL UBC
REACTOME	Degradation of beta-catenin by the destruction complex	82	0.000833	0.01119	PPP2R1A PPP2CA UBC
KEGG	OOCYTE_MEIOSIS	113	0.00012	0.011196	PPP2R1A PPP2CA YWHAG MAP2K1
REACTOME	Meiosis	83	0.000862	0.01152	RPA2 RPA1 HIST2H2BE
REACTOME	G2/M Transition	191	0.000884	0.011741	PPP2R1A PPP2CA YWHAG UBC
REACTOME	Hedgehog 'on' state	85	0.000924	0.011764	ITCH PTCH1 UBC
REACTOME	DNA Replication Pre-Initiation	85	0.000924	0.011764	RPA2 RPA1 UBC
REACTOME	Regulation of PLK1 Activity at G2/M Transition	85	0.000924	0.011834	PPP2R1A YWHAG UBC
REACTOME	M/G1 Transition	85	0.000924	0.011904	RPA2 RPA1 UBC
REACTOME	Mitotic G2-G2/M phases	193	0.000919	0.011975	PPP2R1A PPP2CA YWHAG UBC
REACTOME	Lagging Strand Synthesis	20	0.000917	0.011984	RPA2 RPA1
REACTOME	CTLA4 inhibitory signaling	20	0.000917	0.012021	PPP2R1A PPP2CA
REACTOME	PCNA-Dependent Long Patch Base Excision Repair	21	0.001012	0.012728	RPA2 RPA1
REACTOME	Regulation of signaling by CBL	21	0.001012	0.012728	CBL UBC

REACTOME	Chromosome Maintenance	88	0.001022	0.012783	RPA2 RPA1 HIST2H2BE
REACTOME	Negative regulation of the PI3K/AKT network	89	0.001056	0.013057	TRIB3 PPP2R1A PPP2CA
REACTOME	M Phase	347	0.001056	0.013057	PPP2R1A PPP2CA YWHAG HIST2H2BE UBC
REACTOME	Oxidative Stress Induced Senescence	90	0.001091	0.01341	HIST2H2BE MOV10 UBC
REACTOME	ERK/MAPK targets	22	0.001111	0.013509	PPP2R1A PPP2CA
REACTOME	E2F mediated regulation of DNA replication	22	0.001111	0.013509	PPP2R1A PPP2CA
REACTOME	Signaling by NTRKs	92	0.001162	0.013972	PPP2R1A PPP2CA CLTC
REACTOME	Regulation of TP53 Activity through Phosphorylation	92	0.001162	0.013972	RPA2 RPA1 UBC
REACTOME	DARPP-32 events	24	0.001324	0.015655	PPP2R1A PPP2CA
REACTOME	Telomere C-strand (Lagging Strand) Synthesis	24	0.001324	0.015655	RPA2 RPA1
REACTOME	Anchoring of the basal body to the plasma membrane	96	0.001314	0.01571	IQCB1 PPP2R1A YWHAG
REACTOME	RUNX1 regulates transcription of genes involved in differentiation of HSCs	97	0.001354	0.015922	ITCH HIST2H2BE UBC
REACTOME	Resolution of AP sites via the multiple-nucleotide patch replacement pathway	25	0.001437	0.016629	RPA2 RPA1
REACTOME	Cyclin A/B1/B2 associated events during G2/M transition	25	0.001437	0.016629	PPP2R1A PPP2CA
REACTOME	Nuclear Events (kinase and transcription factor activation)	25	0.001437	0.016718	PPP2R1A PPP2CA
REACTOME	Mitotic Prophase	100	0.001478	0.017011	PPP2R1A PPP2CA HIST2H2BE
REACTOME	Calnexin/calreticulin cycle	26	0.001554	0.017798	PDIA3 UBC
GO	error-prone translesion synthesis	21	1.38E-05	0.018883	RPA2 RPA1 UBC
GO	negative regulation of DNA binding transcription factor activity	65	1.37E-05	0.018883	PHB2 SUMO1 NR0B2 PTCH1
GO	protein localization to chromosome	3	1.48E-05	0.018896	RPA2 RPA1
GO	cytosol	4995	1.14E-05	0.019158	KRT8 CLTC HMGA1 PML IQCB1 PPP2R1A GIPC1 HIST2H2BE

					LATS1 PPP2CA TRIP6 CBL TSR1 TRIB3 ITCH SH3KBP1 CAVIN1 NXF1 RAB7A MAP2K1 MOV10 SOX2 XRCC5 UBC YWHAG
GO	error-free translesion synthesis	20	1.18E-05	0.019158	RPA2 RPA1 UBC
REACTOME	RAF/MAP kinase cascade	228	0.001699	0.019353	PPP2R1A PPP2CA UBC MAP2K1
REACTOME	SUMO E3 ligases SUMOylate target proteins	106	0.001747	0.019789	SUMO1 PML RPA1
REACTOME	Developmental Biology	1023	0.001816	0.020472	KRT8 MAP2K1 CLTC HIST2H2BE SOX2 SH3KBP1 UBC ASH2L
REACTOME	Reproduction	108	0.001843	0.020554	RPA2 RPA1 HIST2H2BE
REACTOME	MAPK1/MAPK3 signaling	233	0.001839	0.020554	PPP2R1A PPP2CA UBC MAP2K1
REACTOME	Signaling by Rho GTPases	402	0.002019	0.022405	PPP2R1A PPP2CA HIST2H2BE CDH1 YWHAG
REACTOME	SUMOylation	112	0.002044	0.022456	SUMO1 PML RPA1
REACTOME	Hedgehog 'off' state	112	0.002044	0.022456	ITCH PTCH1 UBC
REACTOME	Extension of Telomeres	30	0.002069	0.02261	RPA2 RPA1
REACTOME	Signaling by the B Cell Receptor (BCR)	113	0.002097	0.022801	CBL SH3KBP1 UBC
REACTOME	Disassembly of the destruction complex and recruitment of AXIN to the membrane	31	0.002208	0.023425	PPP2R1A PPP2CA
REACTOME	Pre-NOTCH Transcription and Translation	31	0.002208	0.023425	KAT2A MOV10
REACTOME	MAPK targets/ Nuclear events mediated by MAP kinases	31	0.002208	0.02354	PPP2R1A PPP2CA
REACTOME	TGF-beta receptor signaling activates SMADs	31	0.002208	0.023657	CBL UBC
REACTOME	Activated NOTCH1 Transmits Signal to the Nucleus	31	0.002208	0.023774	ITCH UBC
GO	nucleoplasm	3162	2.14E-05	0.023872	RPA2 KRT8 RPA1 TSR1 HIST2H2BE HMGA1 SOX2 ITCH PML XRCC5 SFPQ CAVIN1 NR0B2 UBC IQCB1 ASH2L KAT2A SUMO1 NXF1
GO	nucleotide-excision repair, DNA gap filling	24	2.08E-05	0.023872	RPA2 RPA1 UBC
REACTOME	DNA strand elongation	32	0.002352	0.024829	RPA2 RPA1
BIOCARTA	CHREBP2_PATHWAY	42	0.000115	0.024937	PPP2R1A PPP2CA YWHAG
REACTOME	Synthesis of DNA	119	0.002429	0.025518	RPA2 RPA1 UBC

REACTOME	Activation of the pre-replicative complex	33	0.0025	0.025765	RPA2 RPA1
REACTOME	Oncogene Induced Senescence	33	0.0025	0.025765	MOV10 UBC
REACTOME	Antigen activates B Cell Receptor (BCR) leading to generation of second messengers	33	0.0025	0.025888	CBL SH3KBP1
REACTOME	Post-translational protein modification	1331	0.002495	0.026013	PDIA3 RPA1 HIST2H2BE RAB5C PML UBC KAT2A SUMO1 RAB7A
REACTOME	Negative regulators of DDX58/IFIH1 signaling	34	0.002653	0.027078	ITCH UBC
REACTOME	Signaling by WNT in cancer	34	0.002653	0.027078	PPP2R1A PPP2CA
REACTOME	NOD1/2 Signaling Pathway	35	0.00281	0.02841	ITCH UBC
REACTOME	N-glycan trimming in the ER and Calnexin/Calreticulin cycle	35	0.00281	0.02841	PDIA3 UBC
GO	second-messenger-mediated signaling	4	2.96E-05	0.029351	PPP2R1A PPP2CA
GO	PML body organization	4	2.96E-05	0.029351	PML SUMO1
REACTOME	DNA Replication	127	0.002921	0.029398	RPA2 RPA1 UBC
BIOCARTA	SPRY_PATHWAY	18	0.00074	0.029878	CBL MAP2K1
BIOCARTA	TGFB_PATHWAY	19	0.000826	0.029878	CDH1 MAP2K1
REACTOME	HDR through Single Strand Annealing (SSA)	37	0.003136	0.030986	RPA2 RPA1
REACTOME	Base Excision Repair	37	0.003136	0.030986	RPA2 RPA1
REACTOME	Resolution of Abasic Sites (AP sites)	37	0.003136	0.031128	RPA2 RPA1
REACTOME	Activation of ATR in response to replication stress	37	0.003136	0.031271	RPA2 RPA1
BIOCARTA	TEL_PATHWAY	18	0.00074	0.032126	PPP2CA XRCC5
KEGG	LONG_TERM_DEPRESSION	70	0.000524	0.032511	PPP2R1A PPP2CA MAP2K1
REACTOME	Presynaptic phase of homologous DNA pairing and strand exchange	39	0.003479	0.034221	RPA2 RPA1
GO	nucleotide-excision repair, preincision complex assembly	29	3.73E-05	0.035052	RPA2 RPA1 UBC
REACTOME	PTEN Regulation	138	0.003691	0.036137	PML MOV10 UBC
KEGG	PATHWAYS_IN_CANCER	325	0.000788	0.036635	CBL PML CDH1 PTCH1 MAP2K1
REACTOME	Signaling by ERBB4	41	0.003839	0.037421	ITCH UBC
REACTOME	Homologous DNA Pairing and Strand Exchange	42	0.004025	0.03906	RPA2 RPA1

REACTOME	Beta-catenin independent WNT signaling	143	0.004078	0.039392	CLTC MOV10 UBC
BIOCARTA	PML_PATHWAY	17	0.000659	0.040157	PML SUMO1
BIOCARTA	CBL_PATHWAY	13	0.00038	0.041239	CBL SH3KBP1
REACTOME	TBC/RABGAPs	44	0.00441	0.042226	RAB5C RAB7A
REACTOME	HIV Life Cycle	147	0.004404	0.042226	XRCC5 HMGA1 UBC
REACTOME	Entry of Influenza Virion into Host Cell via Endocytosis	2	0.004495	0.042664	CLTC
REACTOME	Signaling by Hedgehog	148	0.004488	0.042664	ITCH PTCH1 UBC
REACTOME	NOTCH1 Intracellular Domain Regulates Transcription	45	0.004608	0.043549	KAT2A UBC
KEGG	MISMATCH_REPAIR	23	0.001215	0.045209	RPA2 RPA1
REACTOME	Interleukin-3, 5 and GM-CSF signaling	47	0.005018	0.047006	CBL UBC
REACTOME	Pre-NOTCH Expression and Processing	47	0.005018	0.047006	KAT2A MOV10
REACTOME	Signaling by NOTCH3	48	0.005228	0.048769	KAT2A UBC

Supplemental Figure Legend

Figure S1: Efficiency of gene knockdown by inhibitory RNA in pulmonary vascular cell types. In all panels, mean expression of a given target gene transcript in the control group (si-NC) was assigned a fold change of 1, to which corresponding gene expression after gene knockdown was compared. Data are expressed as the mean ± SD (*P < 0.05, **P < 0.01, ***P < 0.001) of 3 independent experiments. Paired samples were compared by 2-tailed Student's t test.

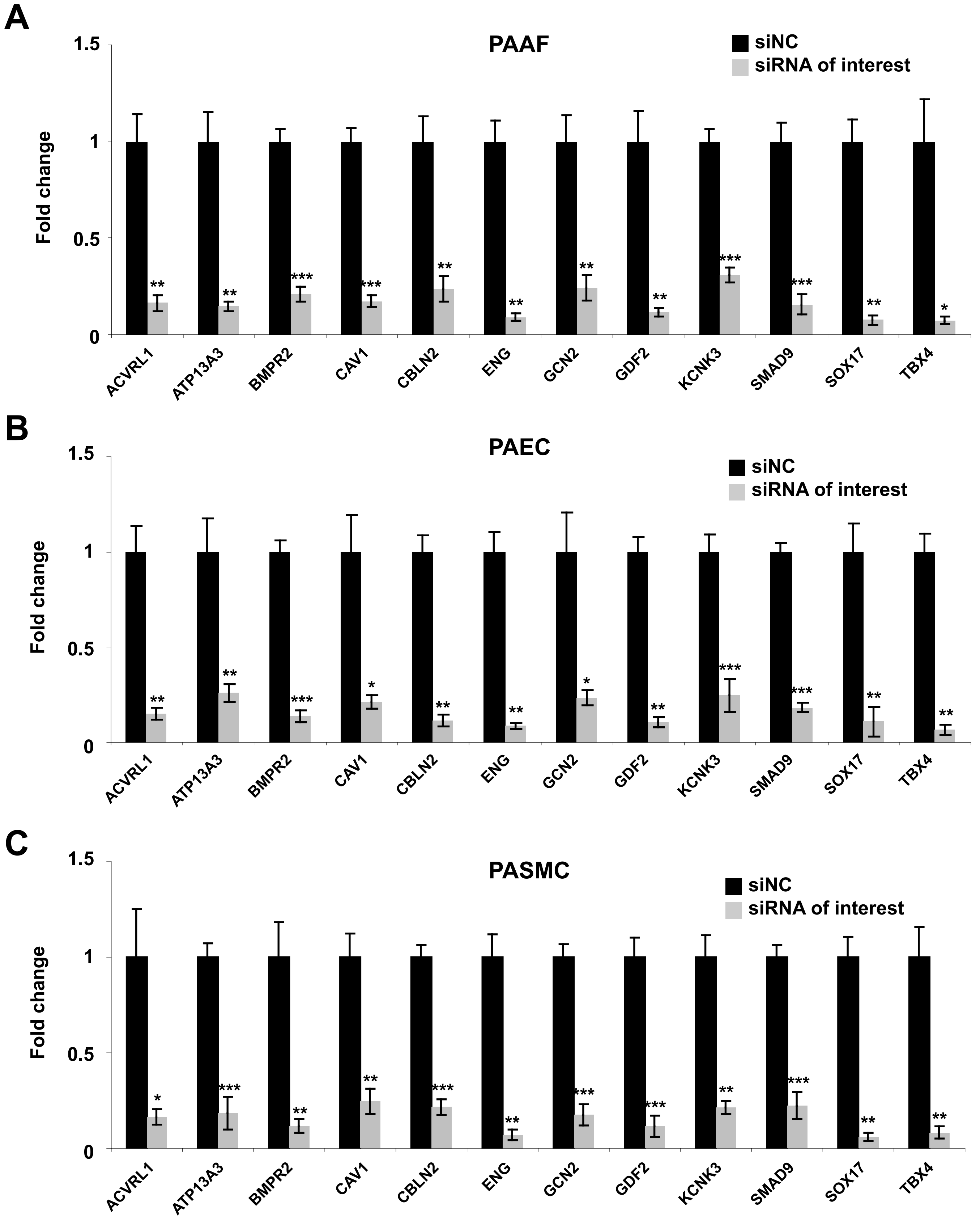
A
PAAF
siNC
siRNA of interest
Fold change
1.5
1
0.5
0
ACVRL1
ATP13A3
BMPR2
CAV1
CBLN2
ENG
GCN2
GDF2
KCNK3
SMAD9
SOX17
TBX4
B
PAEC
siNC
siRNA of interest
Fold change
C
PASMC
siNC
siRNA of interest
Fold change

Figure S1
